# Supplementary material for: Gene Loss and Evolution of the Plastome
Source: Genes (Basel). 2020 Sep 25;11(10):1133. doi: 10.3390/genes11101133 (PMC7650654; doi:10.3390/genes11101133)
Supplement: Supplementary file 1 [file genes-11-01133-s001.zip › Supplementary Materials/Supplementary Data S1.docx]

**Supplementary Data S1**

Chloroplast genomes possess genes that encode at least six different ATP molecules encoded by AtpA, AtpB, AtpE, AtpF, AtpH, and AtpI genes. Among the 2511 analyzed chloroplast genomes, AtpA, AtpB, AtpE, AtpF, AtpH, and AtpI were found to be lost in 8, 8, 12, 14, 13, and 12 species, respectively (Table 2, Supplementary File S4). The loss of Atp genes occurred in algae, eudicots, magnoliids, and monocots, while no loss of Atp genes occurred in any species of bryophytes, pteridophytes, and gymnosperms (Supplementary File S4). The AccD gene in chloroplasts, which encodes the beta-carboxyl transferase subunit of acetyl Co-A carboxylase (ACC) complex, was found to be lost from 387 plant species (Supplementary File S4). The AccD gene was lost in taxa belonging to algae (92 species), eudicots (32 species), gymnosperms (7 species), magnoliids (1 species), monocots (227 species), and protists (27 species), while the AccD gene was found to be present in all bryophytes and pteridophytes.

The CcsA gene in the chloroplast genome encodes a cytochrome C biogenesis protein. CcsA genes were found to be lost in at least 29 species (Table 2). It was lost in taxa belonging to members of algae (eight species), bryophyte (three species), eudicots (six species), magnoliids (one species), monocots (five species), and protists (six species), while no evidence of a loss was observed in members of the pteridophytes and gymnosperms. The CemA gene encodes a chloroplast envelope membrane protein and was found to be lost in 29 species (Table 2). The loss of the CemA gene was found in algae, eudicots, magnoliids, monocots, and protists, while no evidence of deletion was observed in taxa of bryophytes, pteridophytes, and gymnosperms. The ClpP gene encodes an ATP-dependent Clp protease proteolytic subunit that is necessary for ATP hydrolysis. It was observed to be lost in at least 142 species (Supplementary File S4) belonging to the algae (108 species), eudicots (2 species), gymnosperms (3 species), magnoliids (1 species), and protists (28 species). Loss of the ClpP gene was not found in members of bryophytes, pteridophytes, and monocots (Supplementary File S4). The chloroplast genome possesses at least six different Psa genes, PsaA, PsaB, PsaC, PsaI, PsaJ, and PsaM (Table). The PsaA gene was absent in 16 species (three algae, eight eudicots, one magnoliid, and four monocots). PsaB was lost in 10 species (three algae, two eudicots, one magnoliid, and four monocots). PsaC was lost in 19 species (2 algae, 10 eudicots, 1 magnoliid, and 6 monocots). PsaI was absent in 72 species (42 algae, 18 eudicots, 1 magnoliid, 6 monocots, and 5 protists), and PsaJ was lost in 24 species (6 algae, 12 eudicots, 1 gymnosperm, 1 magnoliid, 3 monocots, and 1 protist). The PsaM gene was lost in 2214 species distributed among all of the angiosperm lineages examined in our analysis. The chloroplast encodes 16 different Psb genes (PsbA, PsbB, PsbC, PsbD, PsbE, PsbF, PsbH, PsbI, PsbJ, PsbK, PsbL, PsbM, PsbN, PsbT, PsbZ, and Psb30). The analysis indicated that PsbA, PsbB, PsbC, PsbD, PsbE, PsbF, PsbH, PsbI, PsbJ, PsbK, PsbL, PsbM, PsbN, PsbT, PsbZ, and Psb30 genes were lost in a variety of species. Evidence for the deletion of these genes was observed as follows: PsbA was lost in 12 species (two algae, six eudicots, and four monocots); PsbB was lost in 18 species (2 algae, 11 eudicots, 1 magnoliid, and 4 monocots); PsbC was lost in 16 species (two algae, seven eudicots, one magnoliid, and six monocots); PsbD was lost in 17 species (two algae, nine eudicots, one magnoliid, and five monocots); PsbE and PsbF were lost in 21 species (2 algae, 12 eudicots, 1 magnoliid, and 6 monocots in both cases); PsbH was lost in 20 species (2 algae, 14 eudicots, 1 magnoliid and 3 monocots); PsbI was lost in 18 species (3 algae, 10 eudicots, and 5 monocots); PsbJ was lost in 21 species (2 algae, 12 eudicots, 1 magnoliid, 6 monocots); PsbK was lost in 13 species (two algae, five eudicots, and six monocots); PsbL was lost in 22 species (2 algae, 12 eudicots, 1 magnoliid, 6 monocots, and 1 protist); PsbM was lost in 158 species (115 algae, 9 eudicots, 6 monocots, and 27 protists); PsbN was lost in 22 species (2 algae, 14 eudicots, 1 magnoliid, and 5 monocots); PsbT was lost in 23 species (3 algae, 14 eudicots, and 6 monocots); and PsbZ was lost in 31 species (14 algae, 7 eudicots, 1 magnoliid, 4 monocots, and 5 protists; Supplementary File S4).

The Ndh gene encodes a NAD(P)H-quinone oxidoreductase that shuttles electrons from plastoquinone to quinone in the photosynthetic chain reaction. The analysis indicated that the chloroplast genome encodes at least 11 Ndh genes, NdhA, NdhB, NdhC, NdhD, NdhE, NdhF, NdhG, NdhH, NdhI, NdhJ, and NdhK (Table 2). Evidence for the deletion of these genes was observed as follows: NdhA was lost in: 339 species (207 algae, 1 bryophyte, 35 eudicots, 37 gymnosperms, 2 magnoliids, 27 monocots, 28 protists, and 2 pteridophytes); NdhB was lost in 258 species (211 algae, 5 eudicots, 7 gymnosperms, 1 magnoliid, 3 monocots, 29 protists, and 2 pteridophytes); NdhC was lost in 339 species (212 algae, 38 eudicots, 7 gymnosperms, 2 magnoliids, 49 monocots, 29 protists, and 2 pteridophytes); NdhD was lost in 293 species (214 algae, 28 eudicots, 9 gymnosperms, 1 magnoliid, 9 monocots, 30 protists, and 2 pteridophytes); NdhE was lost in 322 species (218 algae, 30 eudicots, 19 gymnosperms, 1 magnoliid, 20 monocots, 30 protists, 4 pteridophytes); NdhF was lost in 346 species (207 algae, 37 eudicots, 37 gymnosperms, 1 magnoliid, 33 monocots, 30 protists, and 2 pteridophytes); NdhG was lost in 335 species (213 algae, 1 bryophyte, 35 eudicots, 37 gymnosperms, 2 magnoliids, 16 monocots, 30 protists, and 1 pteridophyte); NdhH was lost in 322 species (213 algae, 1 bryophyte, 34 eudicots, 15 gymnosperms, 1 magnoliid, 26 monocots, 30 protists, and 2 pteridophytes); NdhI was lost in 378 species (213 algae, 1 bryophyte, 43 eudicots, 37 gymnosperms, 2 magnoliids, 50 monocots, 30 protists, and 2 pteridophytes); NdhJ was lost in 340 species (215 algae, 40 eudicots, 37 gymnosperms, 2 magnoliids, 15 monocots, 30 protists, and 2 pteridophytes); and NdhK was lost in 331 species (204 algae, 1 bryophyte, 39 eudicots, 7 gymnosperms, 2 magnoliids, 46 monocots, 30 protists, and 2 pteridophytes; Supplementary File S4). The loss of Ndh genes was found to occur in members of algae, bryophytes, pteridophytes, gymnosperms, monocots, eudicots, magnoliids, and protists.

The chloroplast genome encodes PetA (cytochrome f precursor), PetB (cytochrome b6), PetD (cytochrome b6-f complex subunit 4), PetG (cytochrome b6-f complex subunit 5), PetL (cytochrome b6-f complex subunit 6), and PetN (cytochrome b6-f complex subunit 8) genes. Evidence for deletion of these genes was observed as follows: PetA was lost in 33 species (8 algae, 10 eudicot, 1 magnoliid, 6 monocots, and 8 protists); PetB was lost in 15 species (two algae, eight eudicots, one magnoliid, and four monocots); PetD was lost in 36 species (7 algae, 13 eudicots, 1 magnoliid, 6 monocots, and 9 protists); PetL was lost in 71 species (39 algae, 11 eudicots, 1 magnoliid, 4 monocots, and 16 protists); and PetN gene was lost in135 species (106 algae, 5 bryophytes, 11 eudicots, 1 magnoliid, 6 monocots, and 6 protists; Supplementary File S4). PetA was lost in taxa of members of algae, eudicots, magnoliids, monocots, and protists, while PetA was found to be present in bryophytes, pteridophytes, and gymnosperms. PetB gene was found to be lost in taxa of members of algae, eudicots, magnoliids, and monocots, while it was found to be present in bryophytes, pteridophytes, and gymnosperms (Supplementary File S4). PetD was found to be lost in taxa of members of algae, eudicots, magnoliids, monocots, and protists, while it was found to be intact in bryophytes, pteridophytes, and gymnosperms. PetL was found to be lost in taxa of members of algae, eudicots, magnoliids, monocots, and protists, while it was found to be intact in bryophytes, pteridophytes, and gymnosperms. PetN genes were found to be lost in taxa of members of algae, bryophytes, eudicots, magnoliids, monocots, and protists, while it was intact in pteridophytes and gymnosperms.

The chloroplast genome encodes at least nine Rpl genes, Rpl2, Rpl14, Rpl16, Rpl20, Rpl22, Rpl23, Rpl32, Rpl33, and Rpl36 (Table 2). Deletion of these genes was found in taxa of different lineages (Table 2). Rpl2 was lost in two species (one eudicot and one magnoliid). Rpl14 was lost in four species (two algae, one eudicot, and one magnoliid). Rpl16 was lost in three species (two algae and one magnoliid). Rpl22 was lost in 127 species (107 algae, 12 eudicots, 3 magnoliids, 2 monocots, and 3 protists). Rpl32 was lost in 114 species (21 algae, 73 eudicots, 5 gymnosperms, 1 magnoliid, 6 monocots, 8 protists). Rpl33 was lost in 133 species (111 algae, 7 eudicots, 1 magnoliid, 4 monocots, and 10 protists). Rpl23 was lost in 24 species (eight algae, four eudicots, six gymnosperms, one magnoliid, and five monocots; Supplementary File S4).

The chloroplast genome encodes 12 Rps genes, Rps2, Rps3, Rps4, Rps7, Rps8, Rps11, Rps12, Rps14, Rps15, Rps16, Rps18, and Rps19 (Table 2). Our analysis indicated that different Rps genes were lost from a variety of species (Supplementary Table S1). Specifically, Rps2 was lost in three species (one algae, one eudicot, and one magnoliid); Rps3 was lost in three species (two algae and one magnoliid); Rps4 was lost in four species (three algae and one magnoliid); Rps7 was lost in three species (one algae, one gymnosperm, and one magnoliid); Rps8 was lost in three species (one eudicot, one magnoliid, and 1 protist); Rps11 was lost in two species (one eudicot and one magnoliid) and Rps12 was lost in two species (one algae and one magnoliid). The chloroplast genome encodes four Rpo genes, RpoA, RpoB, RpoC1 and RpoC2 (Table 2). RpoA and RpoC1 encode for the alpha-subunit and RpoB and RpoC2 encode the beta-subunit of DNA-dependent RNA polymerase. The analysis revealed the loss of RpoA, RpoB, RpoC1, and RpoC2 genes from the chloroplast genome of several taxa (Supplementary File S4). Specifically, RpoA1 was lost in 26 species (five algae, six bryophytes, seven eudicots, one magnoliid, four monocots, and three protists); RpoB was lost in 19 species (1 algae, 14 eudicots, 1 magnoliid, and 3 monocots); RpoC1 was lost in 21 species (15 eudicots, 1 magnoliid, 5 monocots) and RpoC2 was lost in 13 species (one algae, seven eudicots, one magnoliid, and four monocots). The loss of RpoA occurred across diverse lineages including algae, bryophytes, eudicots, magnoliids, monocots, and protists. Additionally, RpoB was lost in algae, eudicots, magnoliids, and monocots; RpoC1 was lost in eudicots, magnoliids, and monocots and RpoC2 was lost in eudicots, magnoliids, and monocots (Supplementary File S4).

The majority of chloroplast genomes encode four Ycf genes, Ycf1, Ycf2, Ycf3, and Ycf4. Our analysis indicated a dynamic loss of Ycf genes from the chloroplast genome of a variety of taxa (Supplementary File S4). Ycf1 was lost in 161 species (125 algae, 4 eudicots, 1 magnoliid, 3 monocots, and 28 protists), Ycf2 was lost in 219 species (185 algae, 1 eudicot and magnoliid each, 2 monocots, and 30 protists). Ycf3 was lost in 30 species (seven algae, seven eudicots, one magnoliid, six monocots, and nine protists). Ycf4 was lost in 39 species (6 algae, 24 eudicots, 1 magnoliid, 5 monocots, and 3 protists). Although researchers have yet to elucidate the function of Ycf genes, Ycf3 and Ycf4 have been reported to be a photosystem I assembly factor. The loss of Ycf1 and Ycf2 genes was more prominent in algae and the loss of Ycf1 and Ycf2 genes were not found in bryophytes, pteridophytes, and gymnosperms. The loss of Ycf4 was most prominent in eudicots and the loss of Ycf3 and Ycf4 was not observed in bryophytes, pteridophytes, and gymnosperms (Supplementary File S4).
