# Supplementary material for: Gene Loss and Evolution of the Plastome
Source: Genes (Basel). 2020 Sep 25;11(10):1133. doi: 10.3390/genes11101133 (PMC7650654; doi:10.3390/genes11101133)
Supplement: Supplementary file 1 [file genes-11-01133-s001.zip › Supplementary Materials/Supplementary Figure S16.pdf]

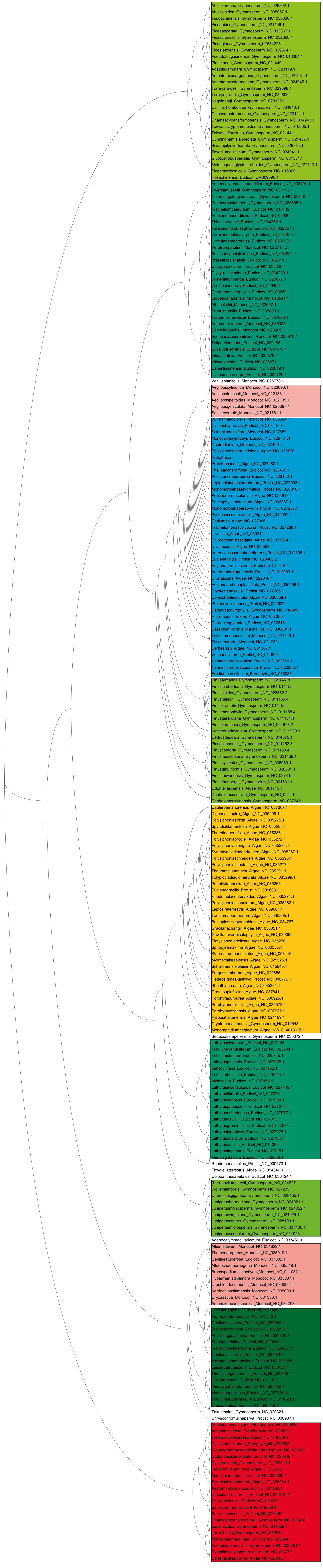

Abieskoreana_Gymnosperm_NC_026892.1
Abiessibirica_Gymnosperm_NC_035067.1
Tsugachinensis_Gymnosperm_NC_030630.1
Piceaabies_Gymnosperm_NC_021456.1
Piceaasperata_Gymnosperm_NC_032367.1
Piceacrassifolia_Gymnosperm_NC_032366.1
Piceaglauca_Gymnosperm_KT634228.1
Piceajezoensis_Gymnosperm_NC_029374.1
Pseudotsugasinensis_Gymnosperm_NC_016064.1
Pinustaeda_Gymnosperm_NC_021440.1
Agathisdammara_Gymnosperm_NC_023119.1
Amentotaxusargotaenia_Gymnosperm_NC_027581.1
Amentotaxusformosana_Gymnosperm_NC_024945.1
Torreyafargesii_Gymnosperm_NC_029398.1
Torreyagrandis_Gymnosperm_NC_034806.1
Nageianagi_Gymnosperm_NC_023120.1
Callitrisrhomboidea_Gymnosperm_NC_034940.1
Calocedrusformosana_Gymnosperm_NC_023121.1
Chamaecyparisformosensis_Gymnosperm_NC_034943.1
Taiwaniacryptomerioides_Gymnosperm_NC_016065.1
Taiwaniaflousiana_Gymnosperm_NC_021441.1
Cunninghamialanceolata_Gymnosperm_NC_021437.1
Sciadopitysverticillata_Gymnosperm_NC_029734.1
Taxodiumdistichum_Gymnosperm_NC_034941.1
Glyptostrobuspensilis_Gymnosperm_NC_031354.1
Metasequoiaglyptostroboides_Gymnosperm_NC_027423.1
Piceamorrisonicola_Gymnosperm_NC_016069.1
Rosachinensis_Eudicot_CM009590.1
Adenocalymmaallamandiflorum_Eudicot_NC_036494.1
Swertiamussotii_Gymnosperm_NC_031155.1
Nothotsugalongibracteata_Gymnosperm_NC_037407.1
Podocarpuslambertii_Gymnosperm_NC_023805.1
Tracheliumcaeruleum_Eudicot_NC_010442.1
Aethionemacordifolium_Eudicot_NC_009265.1
Thlaspiarvense_Eudicot_NC_034362.1
Taraxacumkok-saghyz_Eudicot_NC_032057.1
Taraxacumplatycarpum_Eudicot_NC_031395.1
Utriculariamacrorhiza_Eudicot_NC_025653.1
Veratrumpatulum_Monocot_NC_027715.2
Baccharisgenistelloides_Eudicot_NC_034852.1
Brassaiopsishainla_Eudicot_NC_022811.1
Caraganakozlowii_Eudicot_NC_035228.1
Glycyrrhizalepidota_Eudicot_NC_034229.1
Wisteriafloribunda_Eudicot_NC_027677.1
Wisteriasinensis_Eudicot_NC_029406.1
Caraganamicrophylla_Eudicot_NC_032691.1
Elodeacanadensis_Monocot_NC_018541.1
Albucakirkii_Monocot_NC_032697.1
Prunushumilis_Eudicot_NC_035880.1
Trapamaximowiczii_Eudicot_NC_037023.1
Amomumkrervanh_Monocot_NC_036935.1
Sobraliaboucheri_Monocot_NC_028209.1
Symplocarpusrenifolius_Monocot_NC_033970.1
Taliparitihamabo_Eudicot_NC_030195.1
Eucalyptusgrandis_Eudicot_NC_014570.1
Vitisaceritolia_Eudicot_NC_035878.1
Vitiscoignetiae_Eudicot_NC_036371.1
Camelliasinensis_Eudicot_NC_020019.1
Ulmusmacrocarpa_Eudicot_NC_032720.1
Vanillaplanifolia_Monocot_NC_026778.1
Aegilopscylindrica_Monocot_NC_023096.1
Aegilopstauschii_Monocot_NC_022133.1
Aegilopsspeltoides_Monocot_NC_022135.1
Aegilopsgeniculata_Monocot_NC_023097.1
Secalecereale_Monocot_NC_021761.1
Burmanniadisticha_Monocot_NC_036664.1
Cytinushypocistis_Eudicot_NC_031150.1
Sciaphilaidensiflora_Monocot_NC_027659.1
Monotropahypopitys_Eudicot_NC_029704.1
Gastrodiaelata_Monocot_NC_037409.1
Polysiphoniasertularioides_Algae_NC_035270.1
Prototheca
Protothecacutis_Algae_NC_037480.1
Phelipancheramosa_Eudicot_NC_023465.1
Phelipanchepurpurea_Eudicot_NC_023132.1
Lepidodiniumchlorophorum_Protist_NC_027093.1
Monomorphinaaenigmatica_Protist_NC_020018.1
Prasinodermacoloniale_Algae_NC_024817.1
Palmophyllumcrassum_Algae_NC_033387.1
Monomorphinaparapyrum_Protist_NC_027287.1
Pycnococcusprovasolii_Algae_NC_012097.1
Codiumsp._Algae_NC_037366.1
Trachelomonasvolvocina_Protist_NC_027288.1
Ulvalinza_Algae_NC_030312.1
Chlorodesmisfastigiata_Algae_NC_037364.1
Ulvaflexuosa_Algae_NC_035823.1
Aureococcusanophagefferens_Protist_NC_012898.1
Euglenaviridis_Protist_NC_020460.2
Euglenaformisproxima_Protist_NC_024154.1
Aureoumbralagunensis_Protist_NC_012903.1
Ulvafasciata_Algae_NC_029040.1
Euglenaarchaeoplastidiata_Protist_NC_035156.1
Cryptoglenaskujai_Protist_NC_027286.1
Coleochaetescutata_Algae_NC_030358.1
Phaeocystisglobosa_Protist_NC_021637.1
Cathayaargyrophylla_Gymnosperm_NC_014589.1
Rhipiliapenicilloides_Algae_NC_037365.1
Carnegieagigantea_Eudicot_NC_027618.1
Cassythafiliformis_Magnoliids_NC_036001.1
Triticummonococcum_Monocot_NC_021760.1
Triticumurartu_Monocot_NC_021762.1
Derbesiasp._Algae_NC_031367.1
Vaucherialitorea_Protist_NC_011600.1
Nannochloropsissalina_Protist_NC_022261.1
Nannochloropsisoceanica_Protist_NC_022263.1
Erodiumcarvifolium_Bryophyte_NC_015083.1
Pinusarmandii_Gymnosperm_NC_029847.1
Pinuslambertiana_Gymnosperm_NC_011156.4
Pinussibirica_Gymnosperm_NC_028552.2
Pinusnelsonii_Gymnosperm_NC_011159.4
Pinuskrempfii_Gymnosperm_NC_011155.4
Pinusmonophylla_Gymnosperm_NC_011158.4
Pinusgerardiana_Gymnosperm_NC_011154.4
Pinuskoraiensis_Gymnosperm_NC_004677.2
Keteleeriadavidiana_Gymnosperm_NC_011930.1
Cedrusdeodara_Gymnosperm_NC_014575.1
Piceasitchensis_Gymnosperm_NC_011152.3
Pinuscontorta_Gymnosperm_NC_011153.4
Pinusmassoniana_Gymnosperm_NC_021439.1
Pinussylvestris_Gymnosperm_NC_035069.1
Pinustabuliformis_Gymnosperm_NC_028531.1
Pinustaiwanensis_Gymnosperm_NC_027415.1
Pinusthunbergii_Gymnosperm_NC_001631.1
Odontellasinensis_Algae_NC_001713.1
Cephalotaxusoliveri_Gymnosperm_NC_021110.1
Cephalotaxussinensis_Gymnosperm_NC_037245.1
Caulerpamanorensis_Algae_NC_037367.1
Digeneasimplex_Algae_NC_035298.1
Polysiphoniastricta_Algae_NC_035275.1
Spyridiafilamentosa_Algae_NC_035285.1
Thuretiaquercifolia_Algae_NC_035286.1
Polysiphoniabrodiei_Algae_NC_035272.1
Polysiphoniaelongata_Algae_NC_035274.1
Symphyocladiadendroidea_Algae_NC_035267.1
Polysiphoniaschneideri_Algae_NC_035296.1
Polysiphoniainfestans_Algae_NC_035277.1
Thaumatellaadunca_Algae_NC_035291.1
Tolypiocladiaglomerulata_Algae_NC_035299.1
Periphykonbeckeri_Algae_NC_035261.1
Euglenagracilis_Protist_NC_001603.2
Rhodomelaconfervoides_Algae_NC_035271.1
Polysiphoniascopulorum_Algae_NC_035282.1
Leptosiraterrestris_Algae_NC_009681.1
Taenomaperpusillum_Algae_NC_035295.1
Bulboplastisapyrenoidosa_Algae_NC_034787.1
Gracilariachangii_Algae_NC_038051.1
Gracilariavermiculophylla_Algae_NC_039092.1
Platysiphoniadelicata_Algae_NC_035258.1
Spirogyramaxima_Algae_NC_030355.1
Staurastrumpunctulatum_Algae_NC_008116.1
Myrmeciaisraelensis_Algae_NC_025525.1
Schizomerisleibleinii_Algae_NC_015645.1
Sargassumhorneri_Algae_NC_029856.1
Heterosigmaakashiwo_Protist_NC_010772.1
Sheathiaarcuata_Algae_NC_035231.1
Grateloupiafilicina_Algae_NC_037841.1
Porphyrapurpurea_Algae_NC_000925.1
Porphyraumbilicalis_Algae_NC_035573.1
Porphyrayezoensis_Algae_NC_007932.1
Pyropiahaitanensis_Algae_NC_021189.1
Cryptomeriajaponica_Gymnosperm_NC_010548.1
Monoraphidiumneglectum_Algae_NW_014013626.1
Sequoiasempervirens_Gymnosperm_NC_030372.1
Lathyrusclymenum_Eudicot_NC_027148.1
Trifoliumglanduliferum_Eudicot_NC_025744.1
Trifoliumstrictum_Eudicot_NC_025745.1
Lathyruspalustris_Eudicot_NC_027078.1
Lensculinaris_Eudicot_NC_027152.1
Trifoliumboissieri_Eudicot_NC_025743.1
Viciasativa_Eudicot_NC_027155.1
Lathyrusinconspicuus_Eudicot_NC_027149.1
Lathyruslittoralis_Eudicot_NC_027076.1
Lathyrusvenosus_Eudicot_NC_027080.1
Lathyruspubescens_Eudicot_NC_027079.1
Lathyrusochroleucus_Eudicot_NC_027077.1
Lathyrusdavidii_Eudicot_NC_027073.1
Lathyrusgraminifolius_Eudicot_NC_027074.1
Lathyrusjaponicus_Eudicot_NC_027075.1
Lathyrusodoratus_Eudicot_NC_027150.1
Lathyrussativus_Eudicot_NC_014063.1
Lathyrustingitanus_Eudicot_NC_027151.1
Medicagofalcata_Eudicot_NC_032066.1
Rhodomonassalina_Protist_NC_009573.1
Floydiellaterrestris_Algae_NC_014346.1
Colobanthusapetalus_Eudicot_NC_036424.1
Retrophyllumpiresii_Gymnosperm_NC_024827.1
Wollemianobilis_Gymnosperm_NC_027235.1
Cupressusgigantea_Gymnosperm_NC_028155.1
Juniperusbermudiana_Gymnosperm_NC_024021.1
Juniperusmonosperma_Gymnosperm_NC_024022.1
Juniperusvirginiana_Gymnosperm_NC_024024.1
Juniperuscedrus_Gymnosperm_NC_028190.1
Juniperusmicrosperma_Gymnosperm_NC_037430.1
Juniperusscopulorum_Gymnosperm_NC_024023.1
Adenocalymmadivaricatum_Eudicot_NC_037456.1
Alliumsativum_Monocot_NC_031829.1
Themedaarguens_Monocot_NC_035019.1
Genliseatuberosa_Eudicot_NC_037082.1
Alloeochaeteoreogena_Monocot_NC_035518.1
Brachypodiumdistachyon_Monocot_NC_011032.1
Hyparrheniadiplandra_Monocot_NC_035037.1
Urochloadecumbens_Monocot_NC_030066.1
Kerriochloasiamensis_Monocot_NC_035009.1
Oryzasativa_Monocot_NC_031333.1
Amanakuocangshanica_Monocot_NC_034708.1
Vignaangularis_Eudicot_NC_021091.1
Vignaradiata_Eudicot_NC_013843.1
Genliseaviolacea_Eudicot_NC_037083.1
Stachysbyzantina_Eudicot_NC_029826.1
Phyllostegiavelutina_Eudicot_NC_029820.1
Stenogynebifida_Eudicot_NC_029818.1
Stenogynekanehoana_Eudicot_NC_029821.1
Genliseafiliformis_Eudicot_NC_037079.1
Astragalusmongholicus_Eudicot_NC_029828.1
Lessertiafrutescens_Eudicot_NC_036151.1
Trifoliumlanghanenensis_Eudicot_NC_036109.1
Cicerarietinum_Eudicot_NC_011163.1
Medicagohybrida_Eudicot_NC_027153.1
Medicagopapillosa_Eudicot_NC_027154.1
Trifoliumsubterraneum_Eudicot_NC_011828.1
Cuscutaobtusiflora_Eudicot_NC_009949.1
Taxusmairei_Gymnosperm_NC_020321.1
Chrysochromulinaparva_Protist_NC_036937.1
Ampelopteriselegans_Pteridophyte_NC_035835.1
Athyriumniponicum_Pteridophyte_NC_035839.1
Trebouxiophyceaesp._Algae_NC_018569.1
Diplaziumunbrosum_Bryophyte_NC_035853.1
Stegnogrammasagittifolia_Pteridophyte_NC_035863.1
Codonopsislanceolata_Eudicot_NC_037949.1
Cycasrevoluta_Gymnosperm_NC_020319.1
Klebsormidiumnitens_Algae_DF238762.1
Monsoniamarlothii_Eudicot_NC_029830.1
Symblocharisvandae_Algae_NC_023924.1
Asarumsieboldii_Eudicot_NC_037100.1
Utriculariareniformis_Eudicot_NC_029719.2
Salixbabylonica_Eudicot_NC_028350.1
Salixpurpurea_Eudicot_KP019639.1
Salixsuchowensis_Eudicot_NC_026462.1
Chamaecyparishodginsii_Gymnosperm_NC_036996.1
Larixdecidua_Gymnosperm_NC_016058.1
Larixsibirica_Gymnosperm_NC_036811.1
Monsoniamarginata_Eudicot_NC_029694.1
Pseudolarixamabilis_Gymnosperm_NC_030631.1
Cyanidioschyzonmerolae_Algae_NC_004799.1
Carteriacerasiformis_Algae_NC_026585.1
