# Supplementary material for: Gene Loss and Evolution of the Plastome
Source: Genes (Basel). 2020 Sep 25;11(10):1133. doi: 10.3390/genes11101133 (PMC7650654; doi:10.3390/genes11101133)
Supplement: Supplementary file 1 [file genes-11-01133-s001.zip › Supplementary Materials/Supplementary Figures.pptx]

## Slide 1
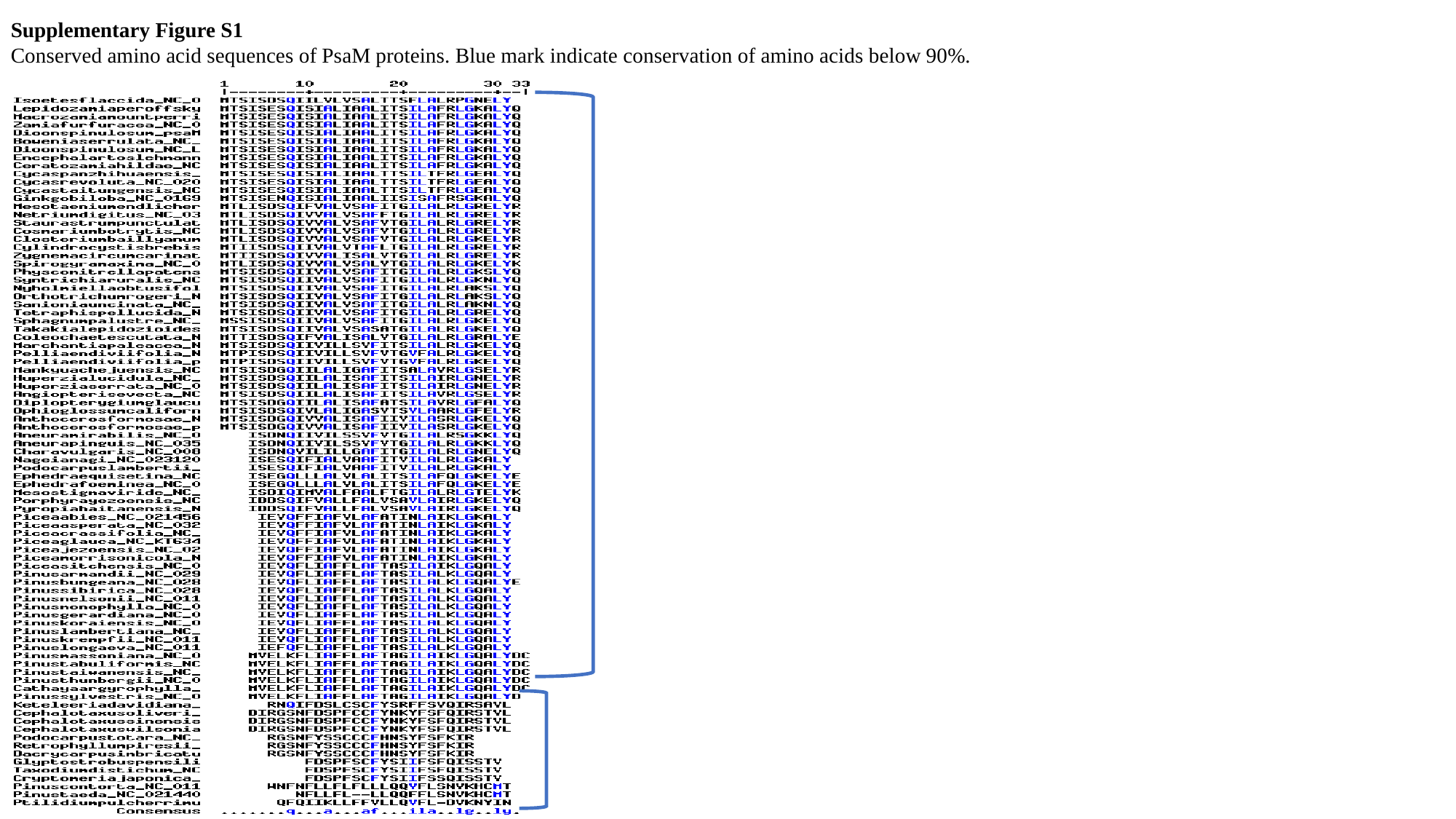

Supplementary Figure S1
Conserved amino acid sequences of PsaM proteins. Blue mark indicate conservation of amino acids below 90%.

## Slide 2
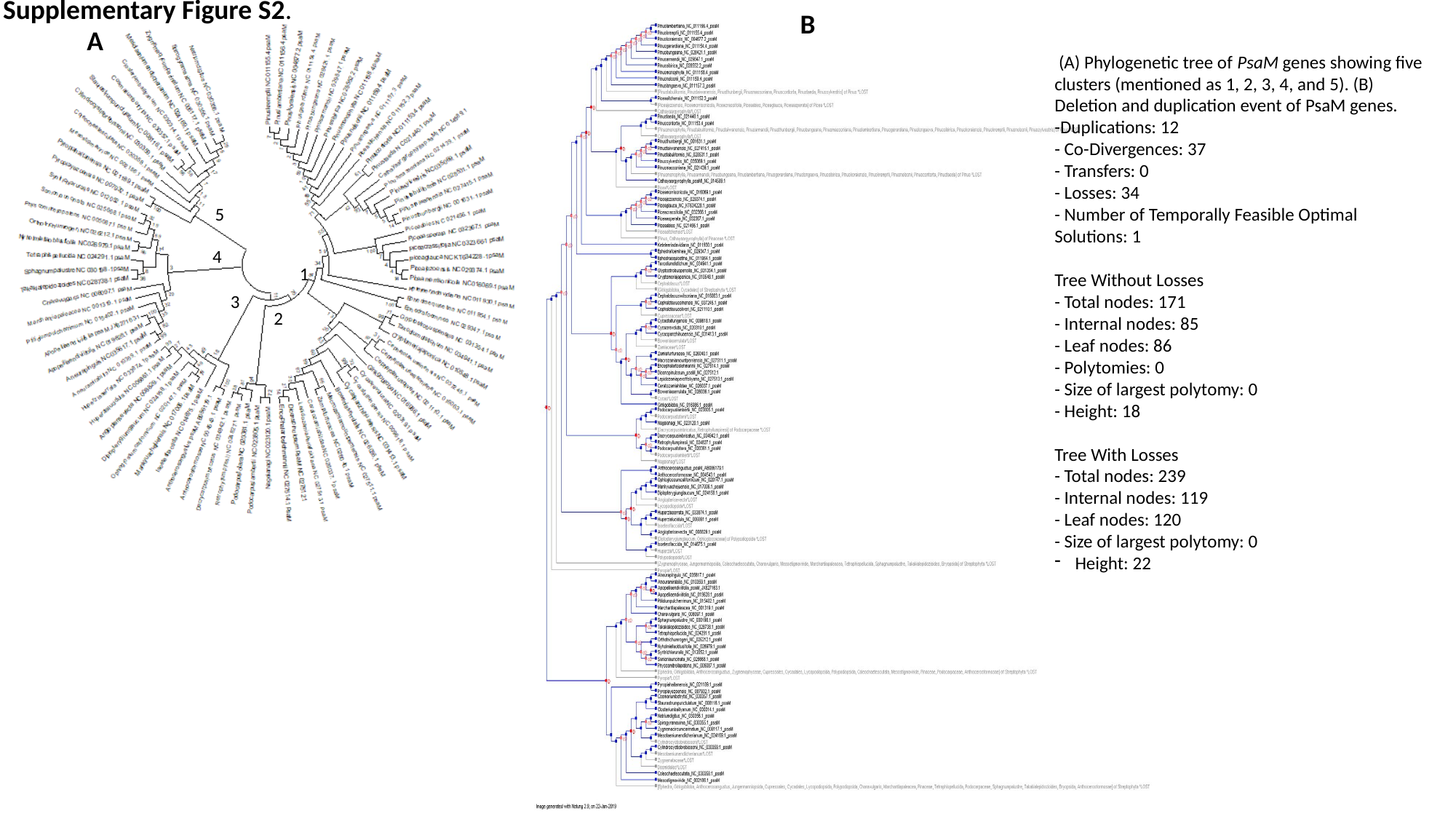

Supplementary Figure S2.
B
A
 (A) Phylogenetic tree of PsaM genes showing five clusters (mentioned as 1, 2, 3, 4, and 5). (B)
Deletion and duplication event of PsaM genes.
-Duplications: 12
- Co-Divergences: 37
- Transfers: 0
- Losses: 34
- Number of Temporally Feasible Optimal Solutions: 1
Tree Without Losses
- Total nodes: 171
- Internal nodes: 85
- Leaf nodes: 86
- Polytomies: 0
- Size of largest polytomy: 0
- Height: 18
Tree With Losses
- Total nodes: 239
- Internal nodes: 119
- Leaf nodes: 120
- Size of largest polytomy: 0
Height: 22
5
4
1
3
2

## Slide 3
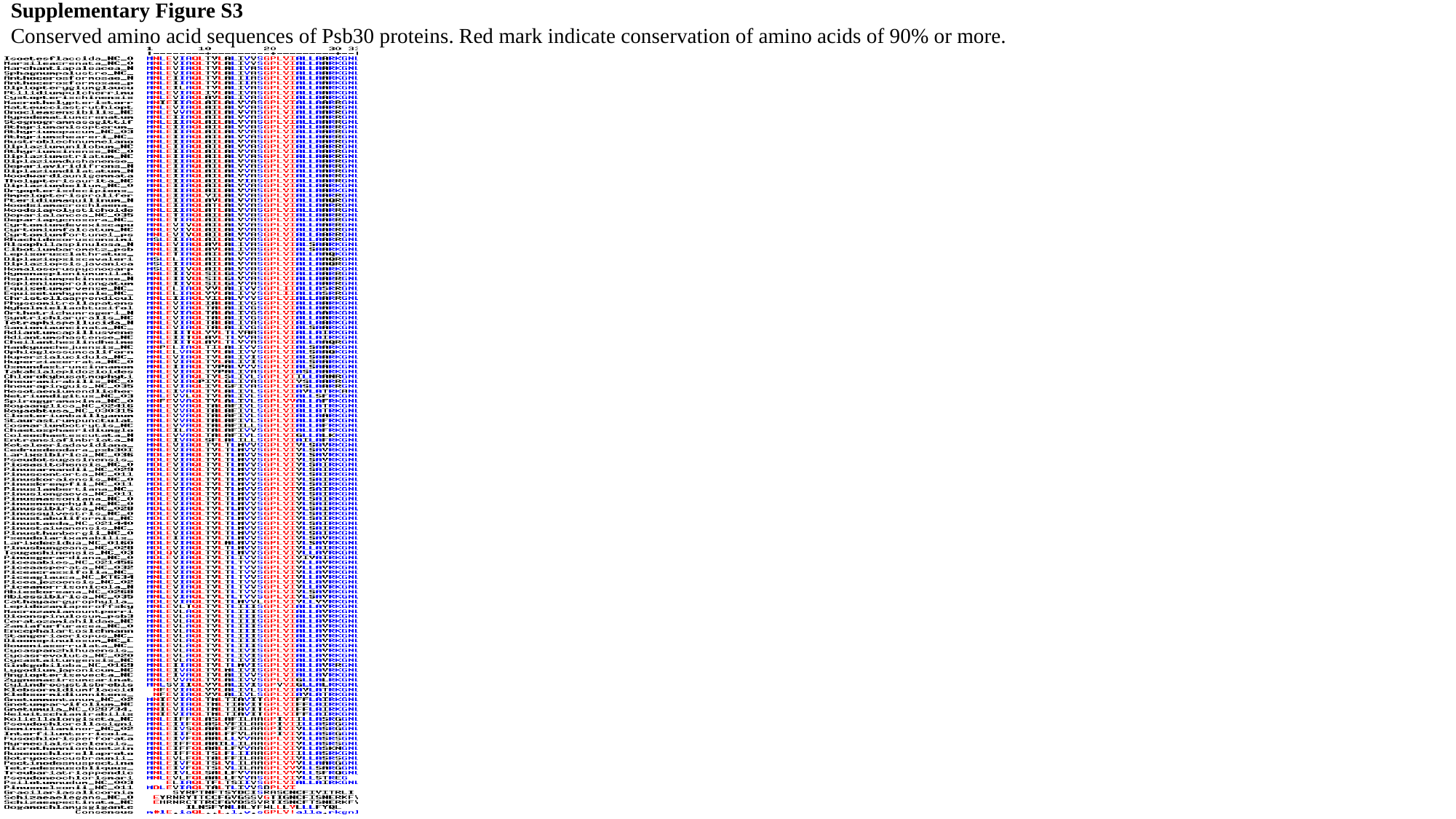

Supplementary Figure S3
Conserved amino acid sequences of Psb30 proteins. Red mark indicate conservation of amino acids of 90% or more.

## Slide 4
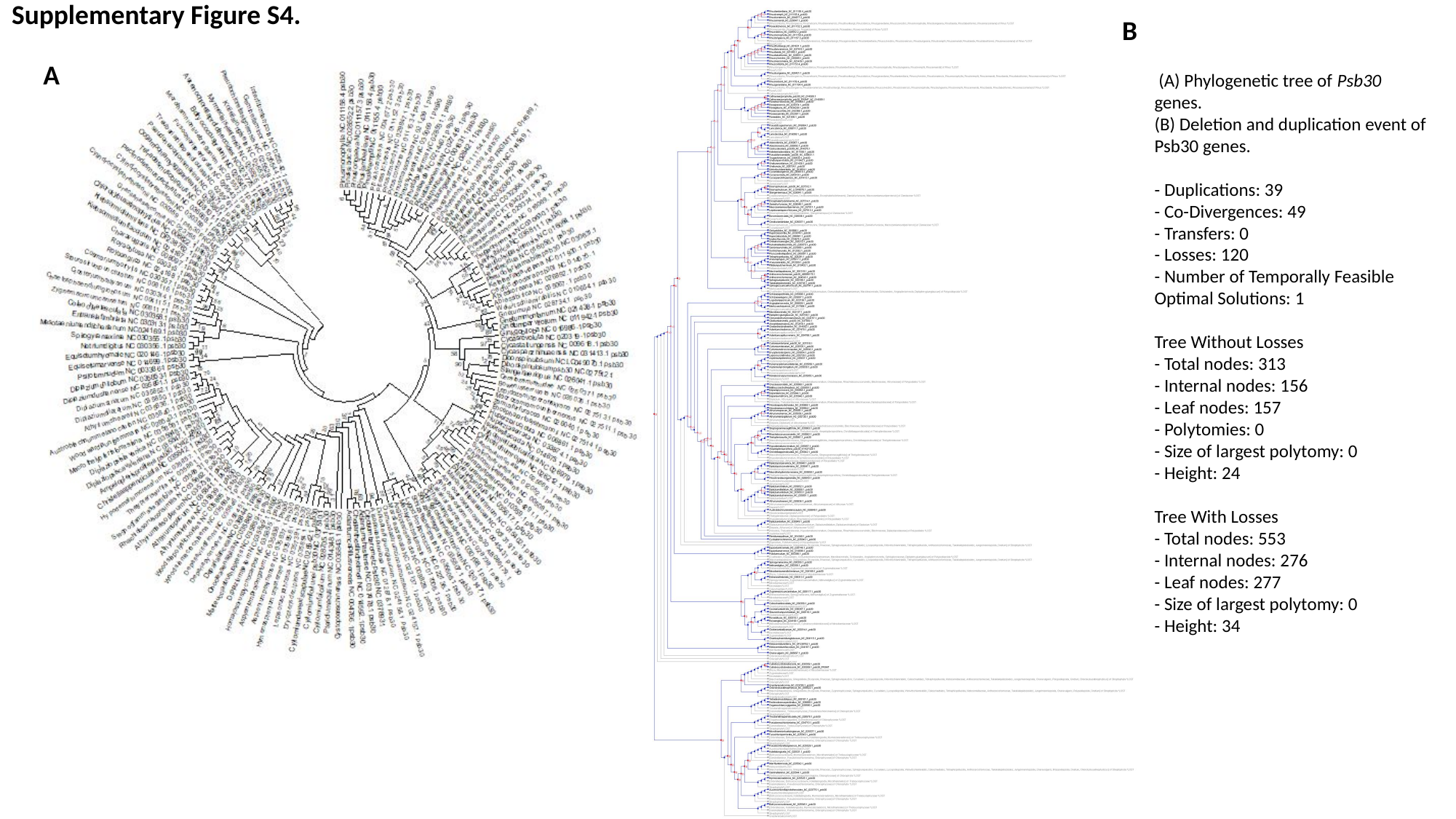

Supplementary Figure S4.
B
A
 (A) Phylogenetic tree of Psb30 genes.
(B) Deletion and duplication event of Psb30 genes.
- Duplications: 39
- Co-Divergences: 49
- Transfers: 0
- Losses: 120
- Number of Temporally Feasible Optimal Solutions: 1
Tree Without Losses
- Total nodes: 313
- Internal nodes: 156
- Leaf nodes: 157
- Polytomies: 0
- Size of largest polytomy: 0
- Height: 24
Tree With Losses
- Total nodes: 553
- Internal nodes: 276
- Leaf nodes: 277
- Size of largest polytomy: 0
- Height: 34

## Slide 5
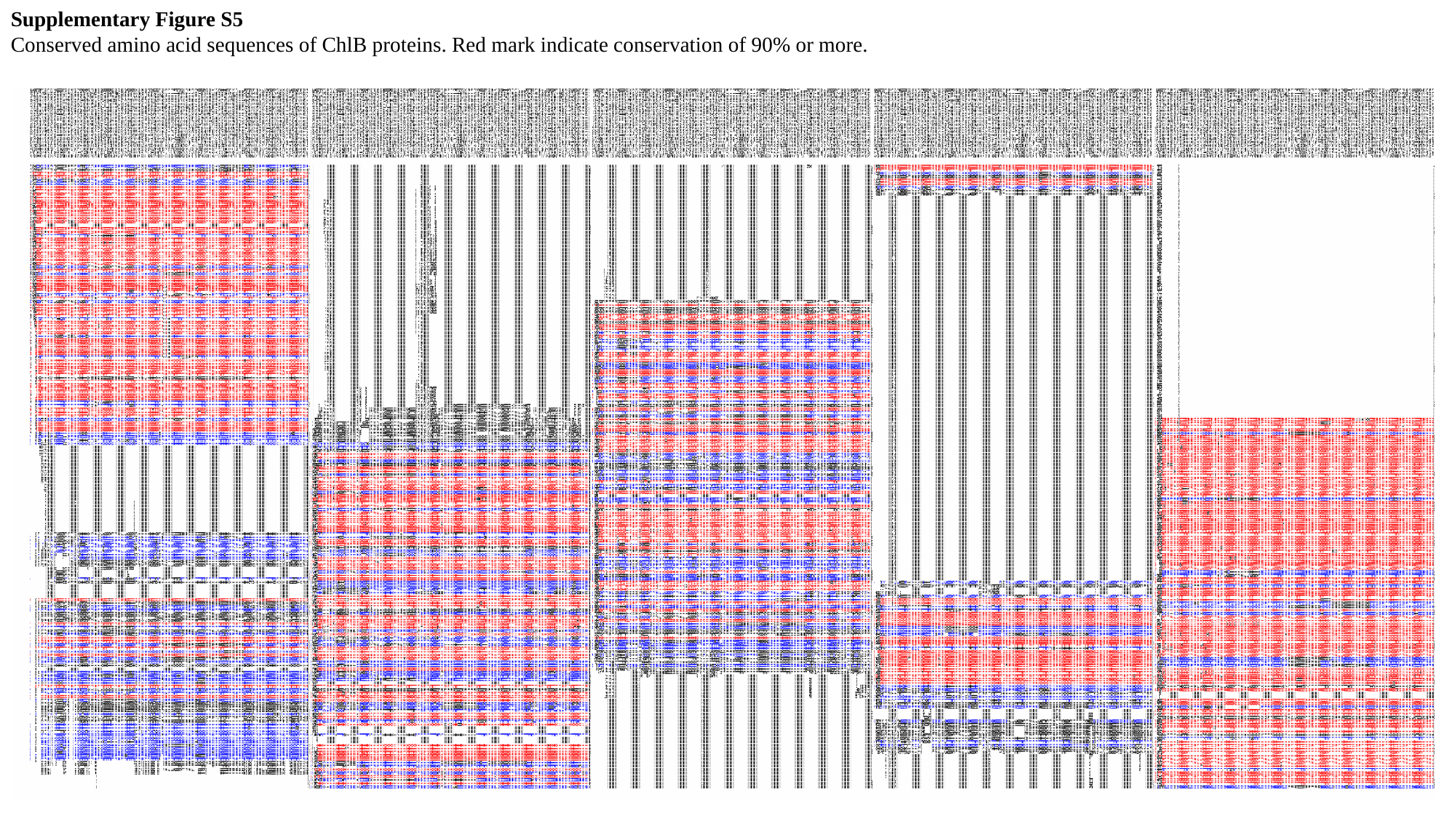

Supplementary Figure S5
Conserved amino acid sequences of ChlB proteins. Red mark indicate conservation of 90% or more.

## Slide 6
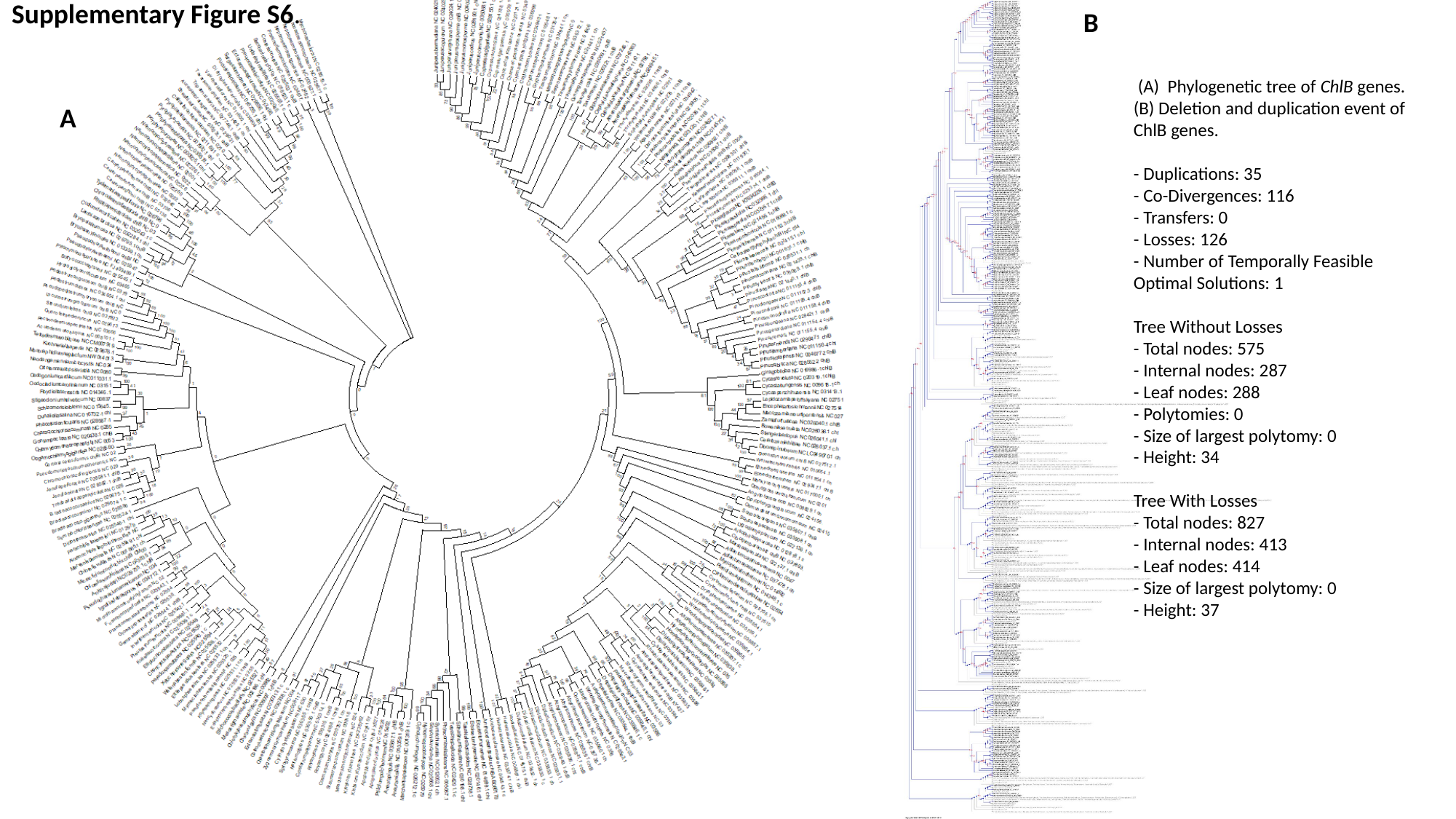

B
 (A) Phylogenetic tree of ChlB genes. (B) Deletion and duplication event of ChlB genes.
- Duplications: 35
- Co-Divergences: 116
- Transfers: 0
- Losses: 126
- Number of Temporally Feasible Optimal Solutions: 1
Tree Without Losses
- Total nodes: 575
- Internal nodes: 287
- Leaf nodes: 288
- Polytomies: 0
- Size of largest polytomy: 0
- Height: 34
Tree With Losses
- Total nodes: 827
- Internal nodes: 413
- Leaf nodes: 414
- Size of largest polytomy: 0
- Height: 37
A
Supplementary Figure S6.

## Slide 7
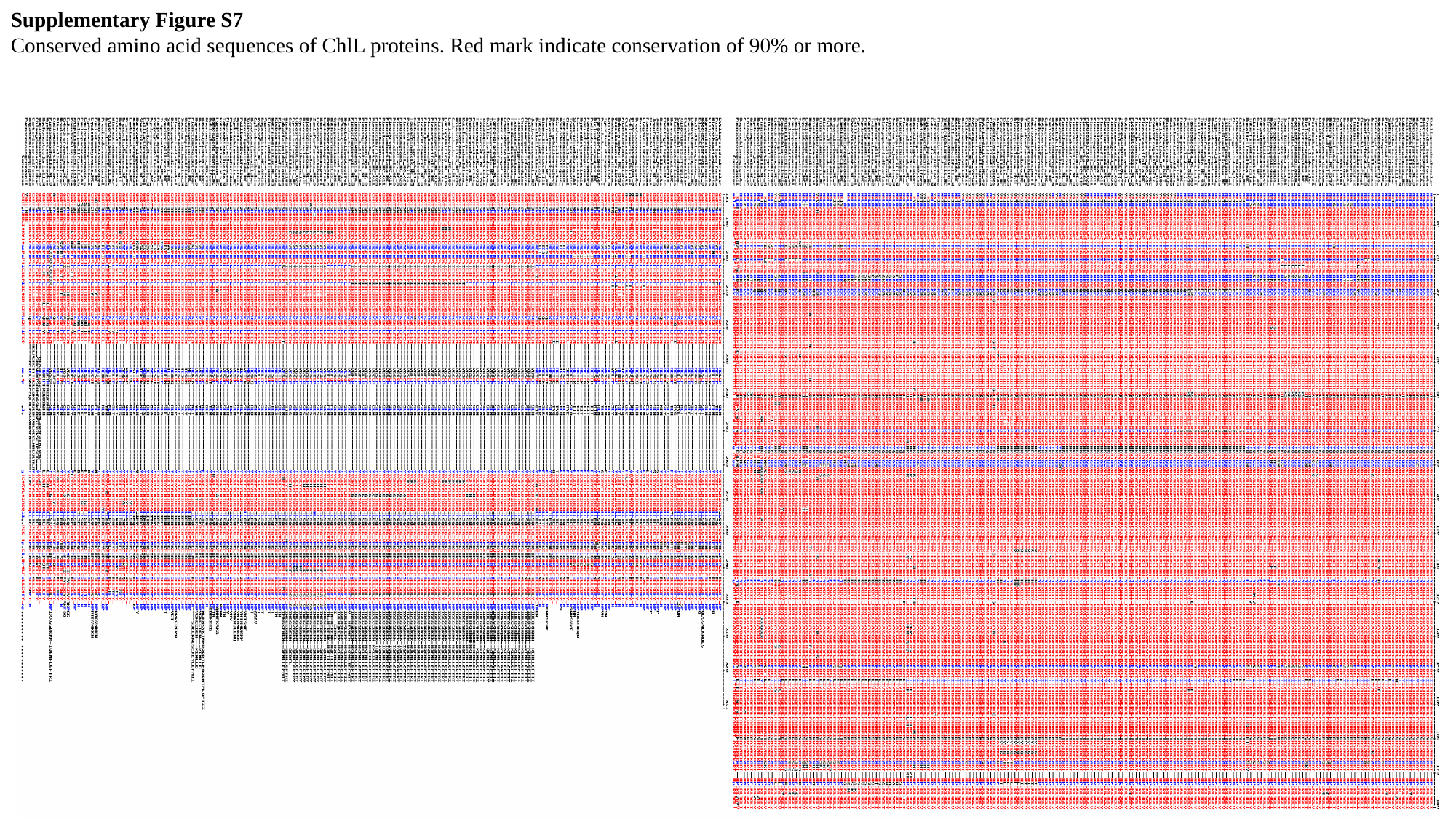

Supplementary Figure S7
Conserved amino acid sequences of ChlL proteins. Red mark indicate conservation of 90% or more.

## Slide 8
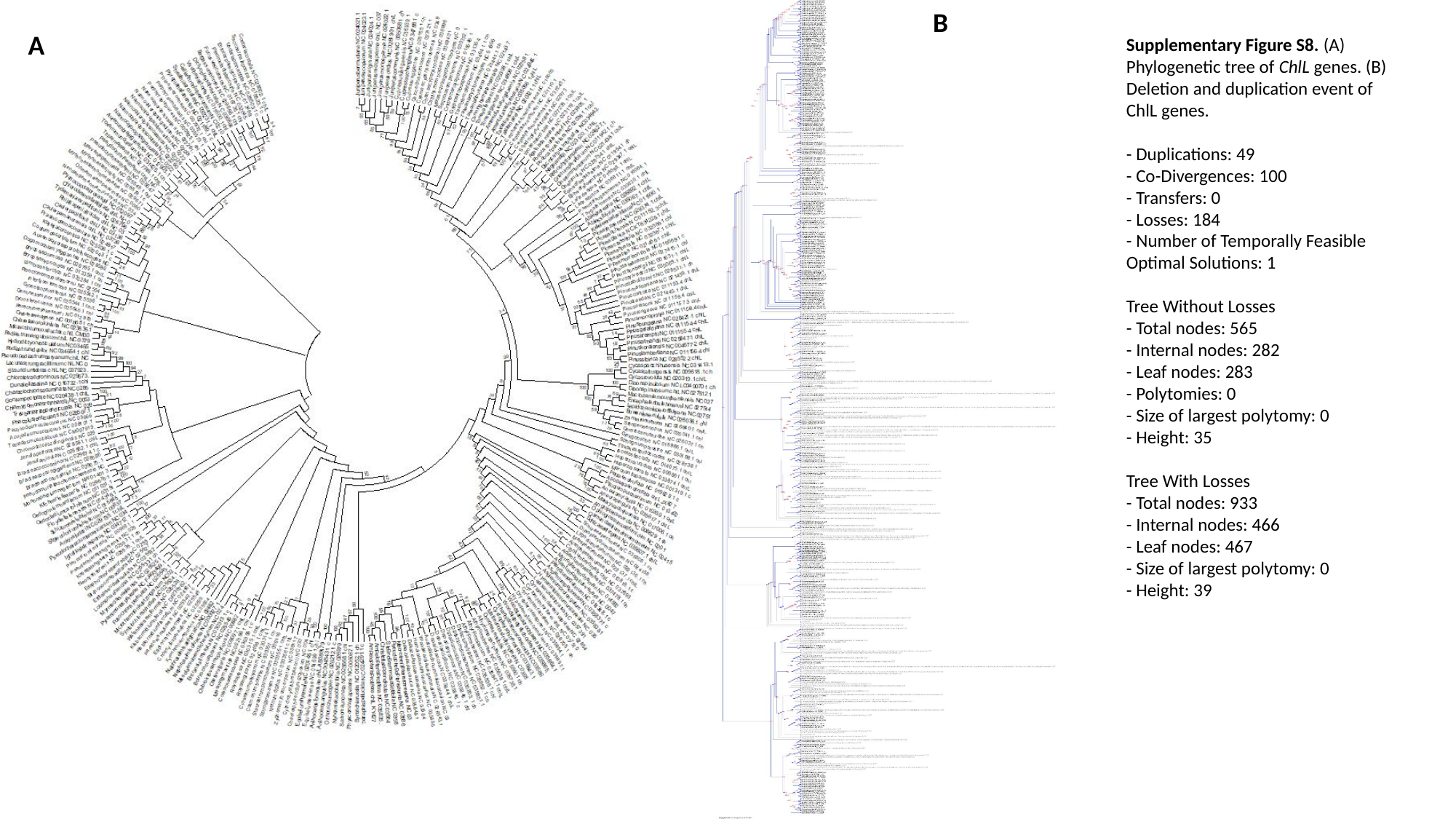

B
A
Supplementary Figure S8. (A) Phylogenetic tree of ChlL genes. (B) Deletion and duplication event of ChlL genes.
- Duplications: 49
- Co-Divergences: 100
- Transfers: 0
- Losses: 184
- Number of Temporally Feasible Optimal Solutions: 1
Tree Without Losses
- Total nodes: 565
- Internal nodes: 282
- Leaf nodes: 283
- Polytomies: 0
- Size of largest polytomy: 0
- Height: 35
Tree With Losses
- Total nodes: 933
- Internal nodes: 466
- Leaf nodes: 467
- Size of largest polytomy: 0
- Height: 39

## Slide 9
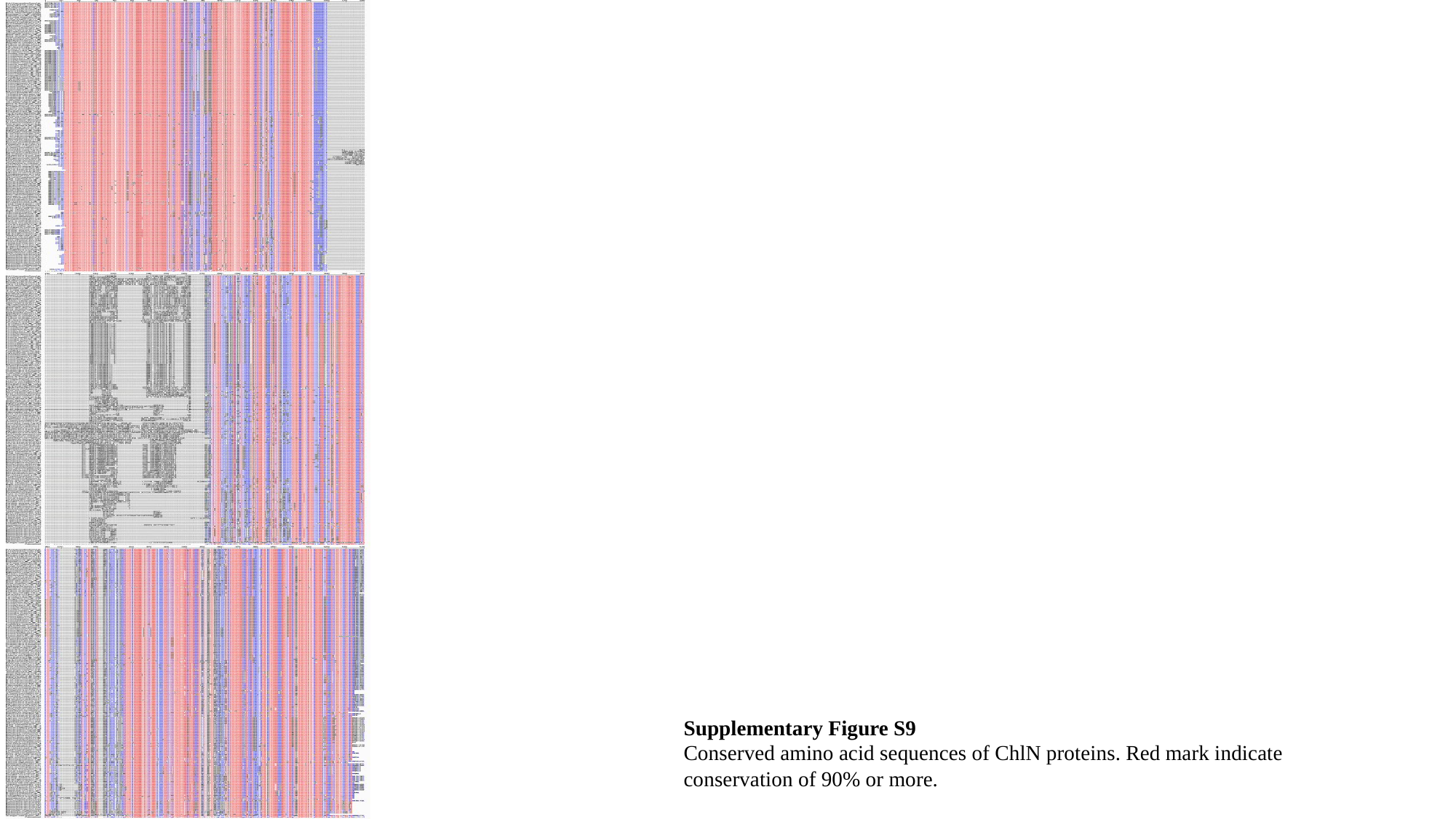

Supplementary Figure S9
Conserved amino acid sequences of ChlN proteins. Red mark indicate conservation of 90% or more.

## Slide 10
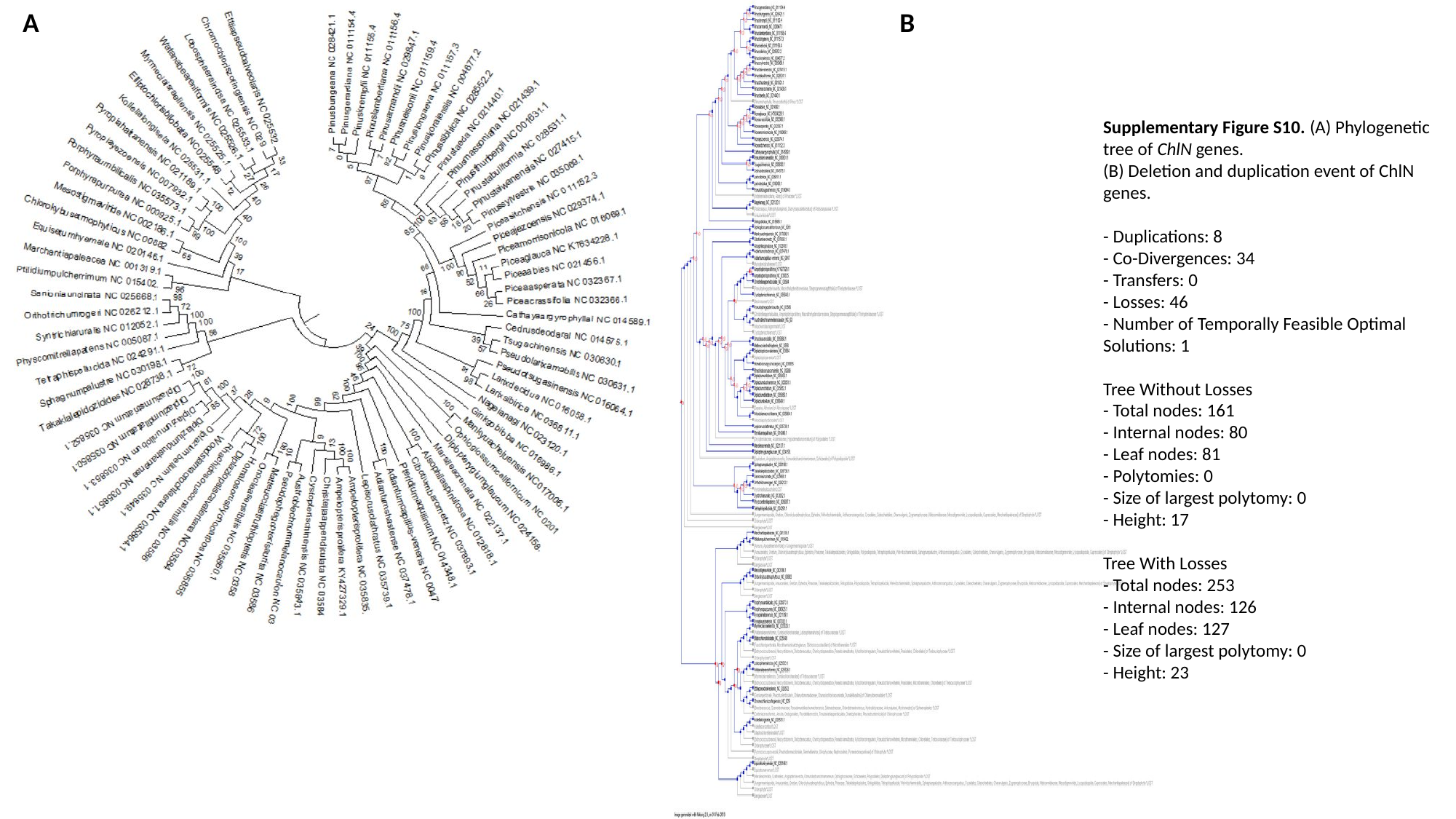

A
B
Supplementary Figure S10. (A) Phylogenetic tree of ChlN genes.
(B) Deletion and duplication event of ChlN genes.
- Duplications: 8
- Co-Divergences: 34
- Transfers: 0
- Losses: 46
- Number of Temporally Feasible Optimal Solutions: 1
Tree Without Losses
- Total nodes: 161
- Internal nodes: 80
- Leaf nodes: 81
- Polytomies: 0
- Size of largest polytomy: 0
- Height: 17
Tree With Losses
- Total nodes: 253
- Internal nodes: 126
- Leaf nodes: 127
- Size of largest polytomy: 0
- Height: 23

## Slide 11
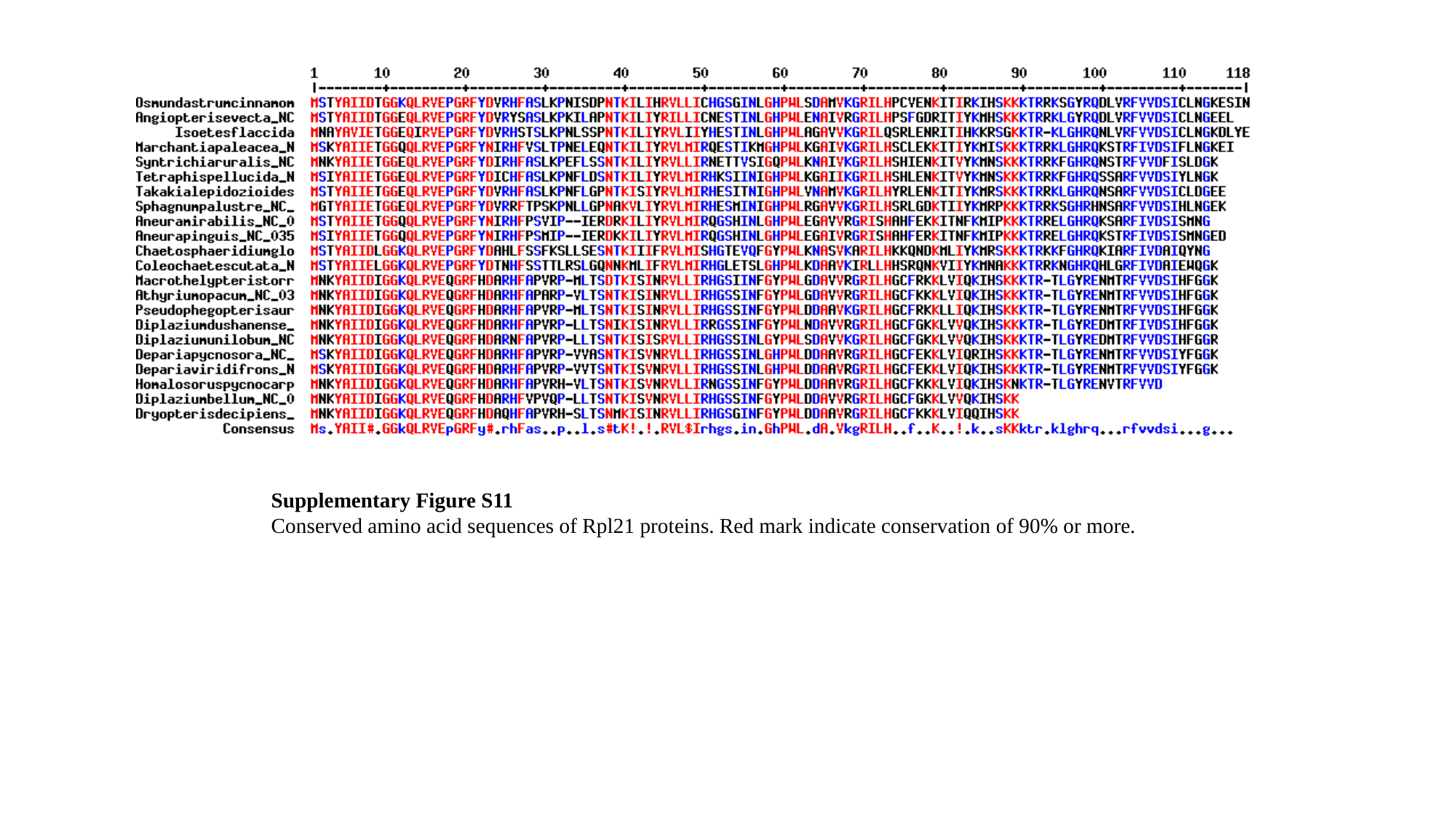

Supplementary Figure S11
Conserved amino acid sequences of Rpl21 proteins. Red mark indicate conservation of 90% or more.

## Slide 12
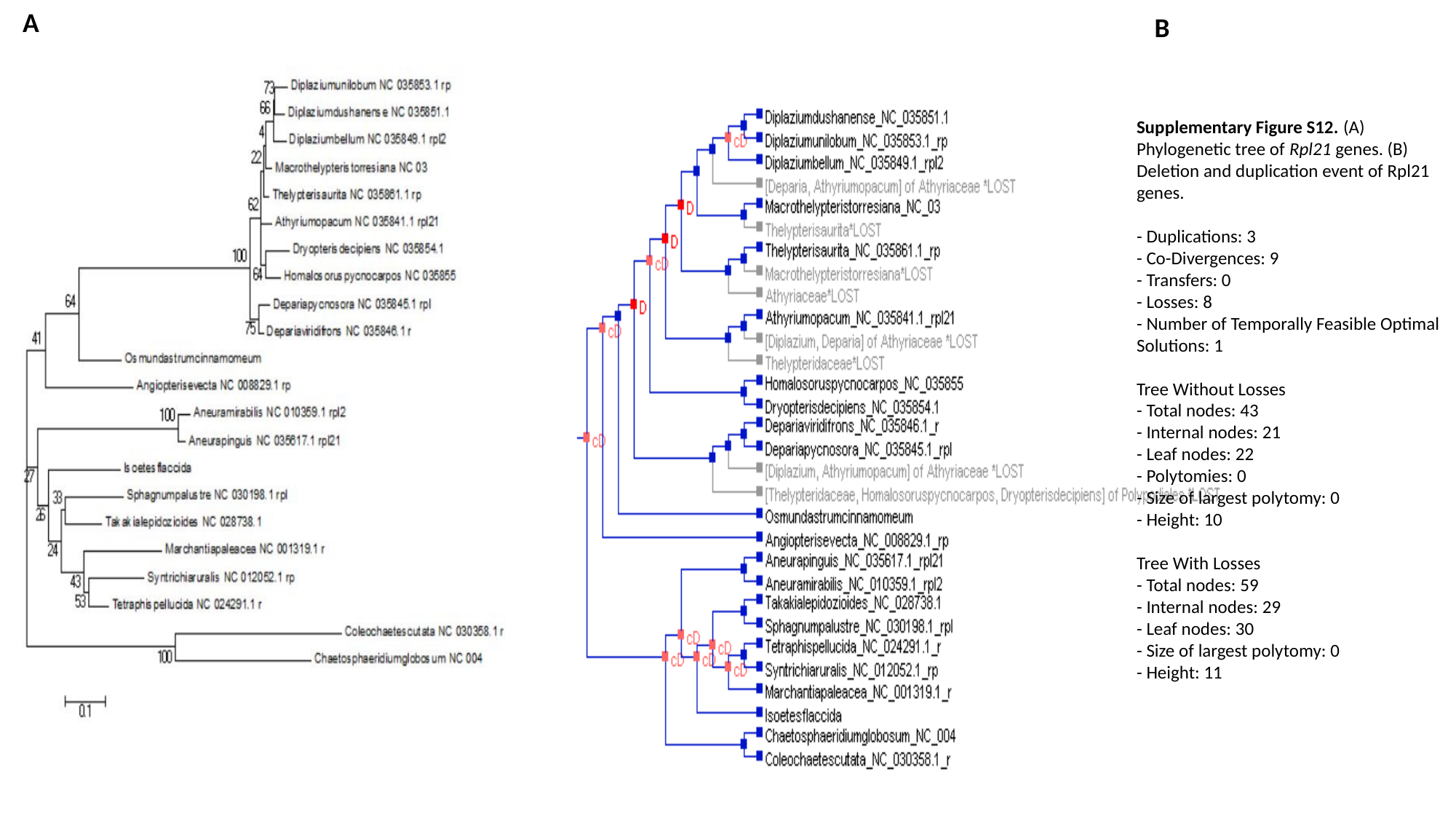

A
B
Supplementary Figure S12. (A) Phylogenetic tree of Rpl21 genes. (B) Deletion and duplication event of Rpl21 genes.
- Duplications: 3
- Co-Divergences: 9
- Transfers: 0
- Losses: 8
- Number of Temporally Feasible Optimal Solutions: 1
Tree Without Losses
- Total nodes: 43
- Internal nodes: 21
- Leaf nodes: 22
- Polytomies: 0
- Size of largest polytomy: 0
- Height: 10
Tree With Losses
- Total nodes: 59
- Internal nodes: 29
- Leaf nodes: 30
- Size of largest polytomy: 0
- Height: 11

## Slide 13
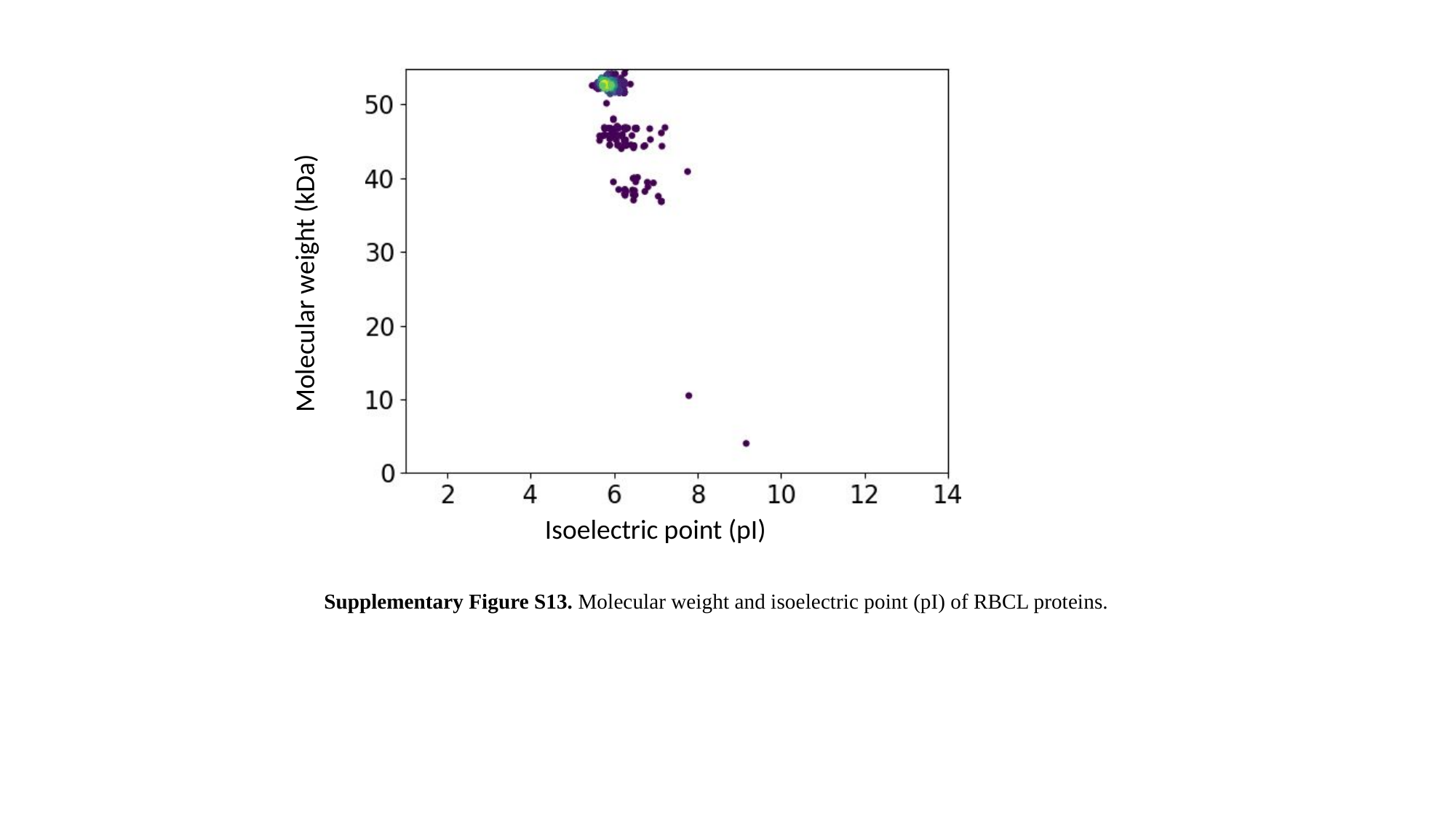

Molecular weight (kDa)
Isoelectric point (pI)
Supplementary Figure S13. Molecular weight and isoelectric point (pI) of RBCL proteins.

## Slide 14
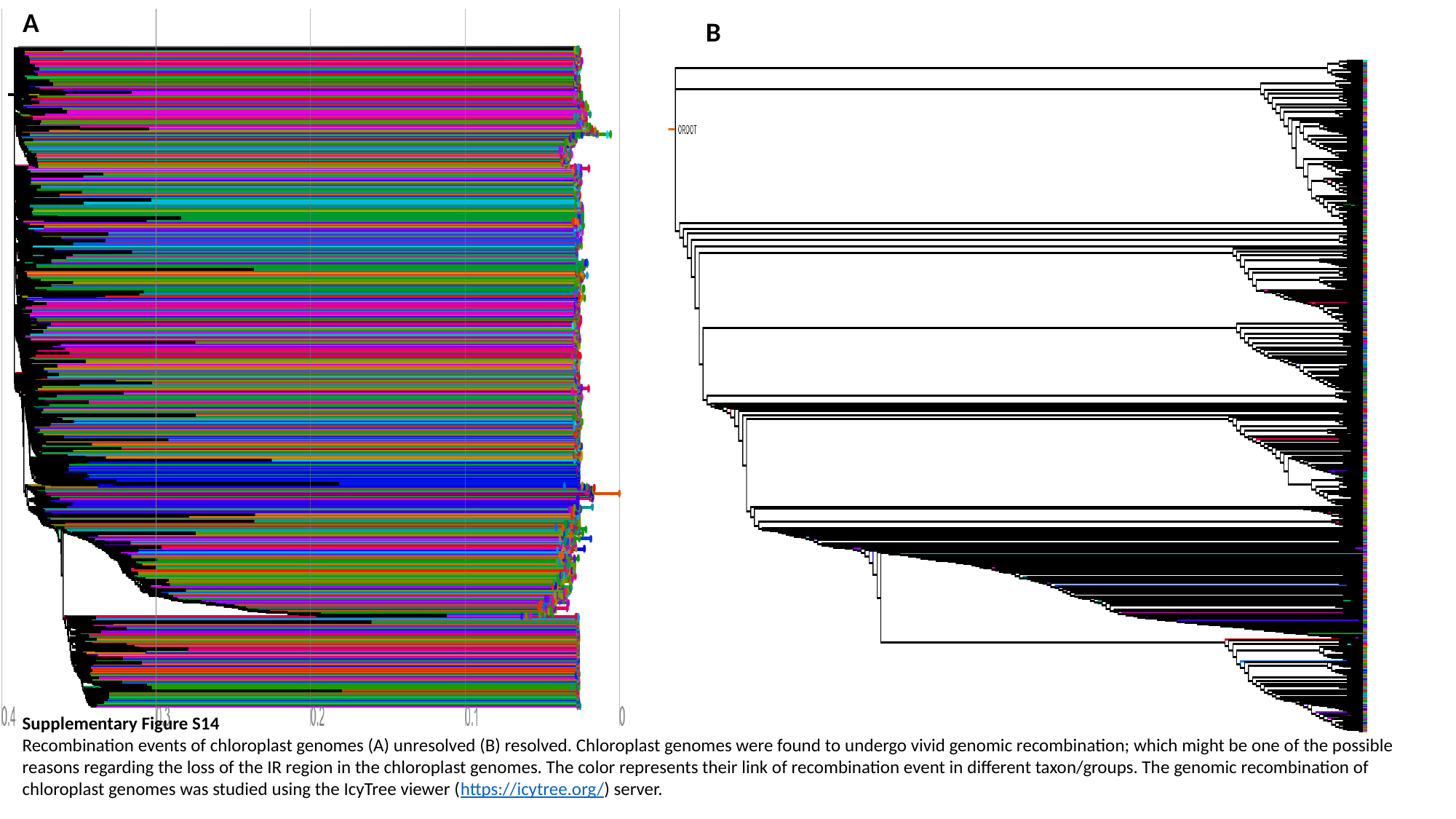

A
B
Supplementary Figure S14
Recombination events of chloroplast genomes (A) unresolved (B) resolved. Chloroplast genomes were found to undergo vivid genomic recombination; which might be one of the possible reasons regarding the loss of the IR region in the chloroplast genomes. The color represents their link of recombination event in different taxon/groups. The genomic recombination of chloroplast genomes was studied using the IcyTree viewer (https://icytree.org/) server.

## Slide 15
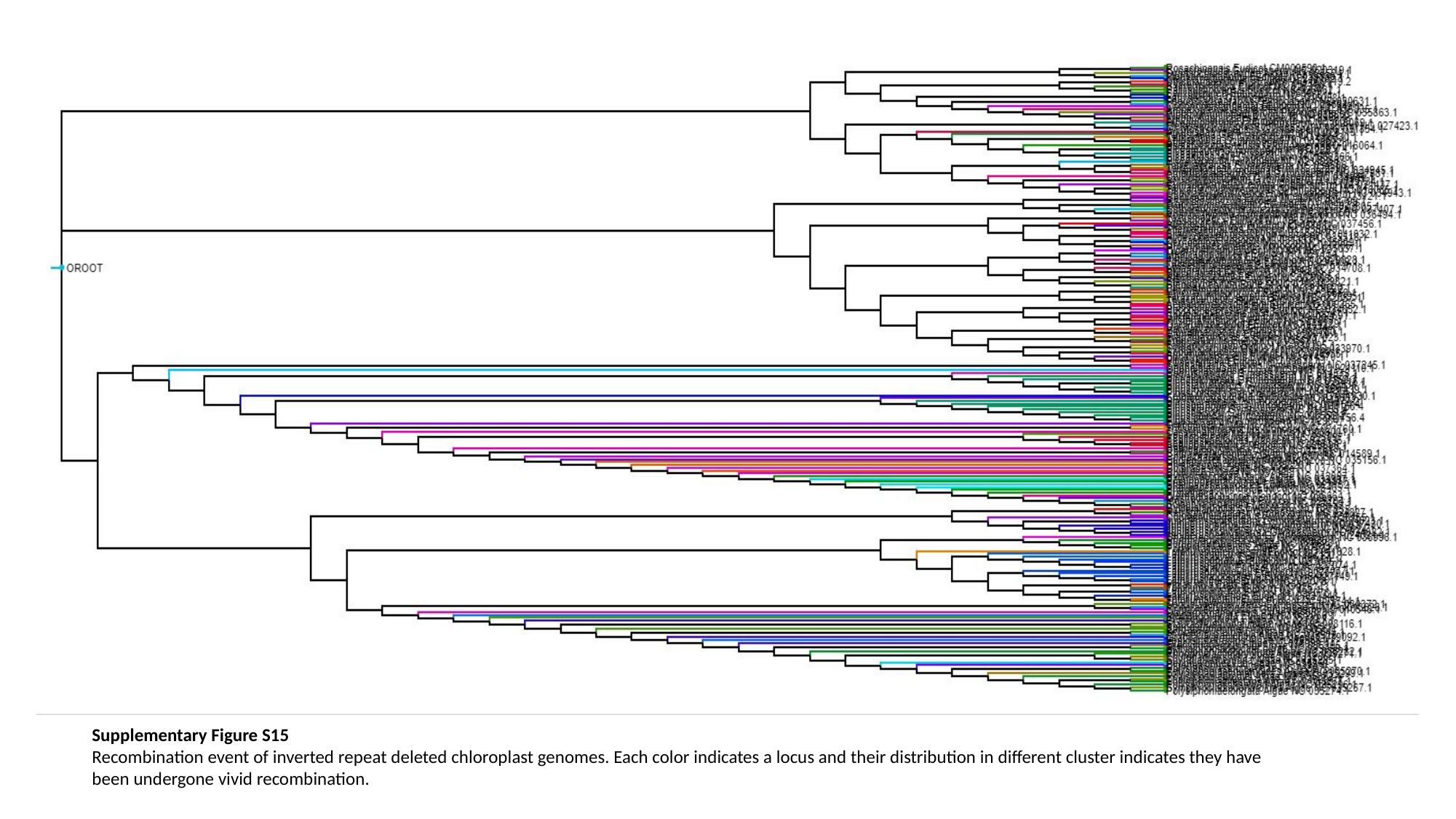

Supplementary Figure S15
Recombination event of inverted repeat deleted chloroplast genomes. Each color indicates a locus and their distribution in different cluster indicates they have been undergone vivid recombination.

## Slide 16
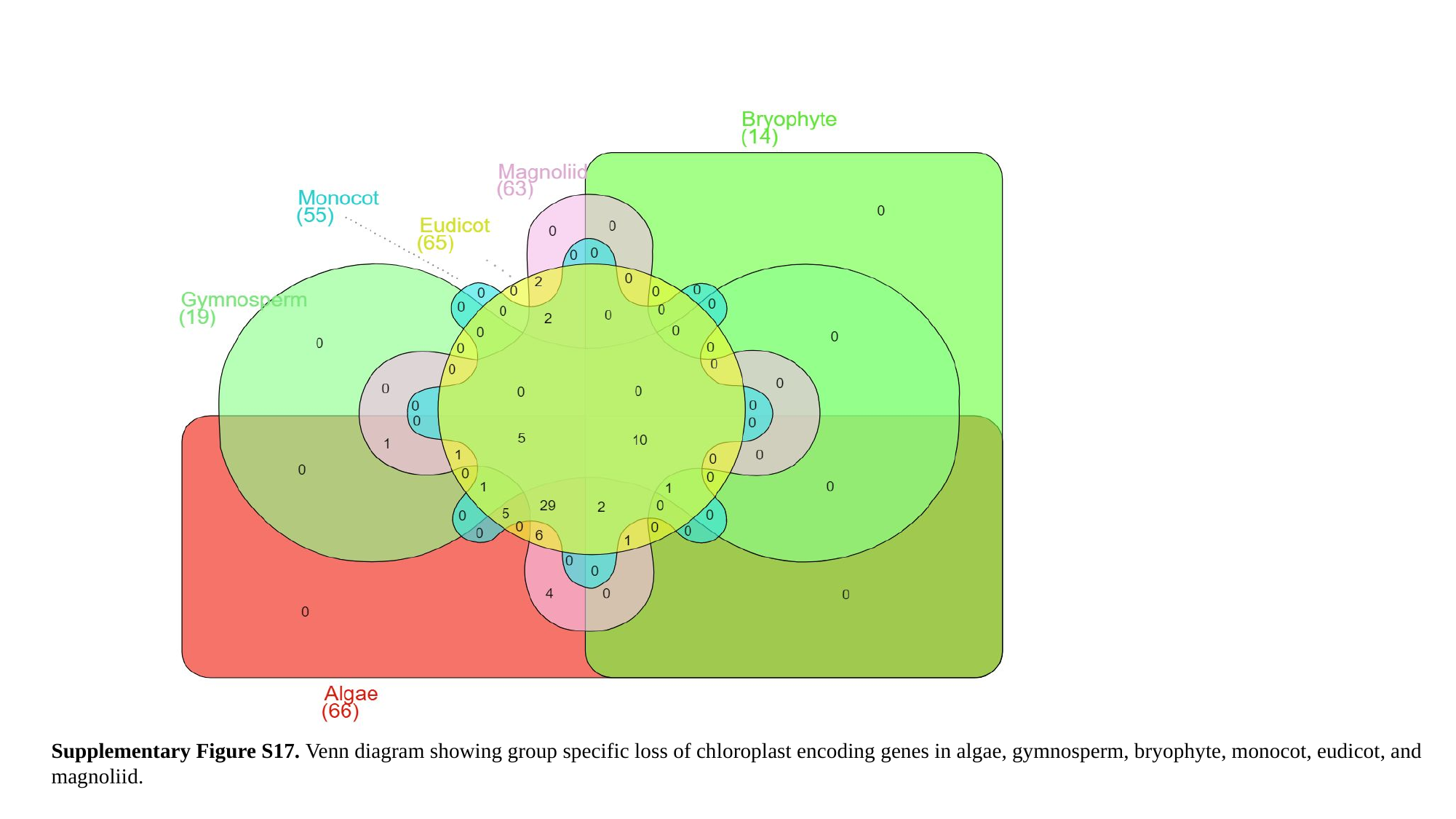

Supplementary Figure S17. Venn diagram showing group specific loss of chloroplast encoding genes in algae, gymnosperm, bryophyte, monocot, eudicot, and magnoliid.

## Slide 17
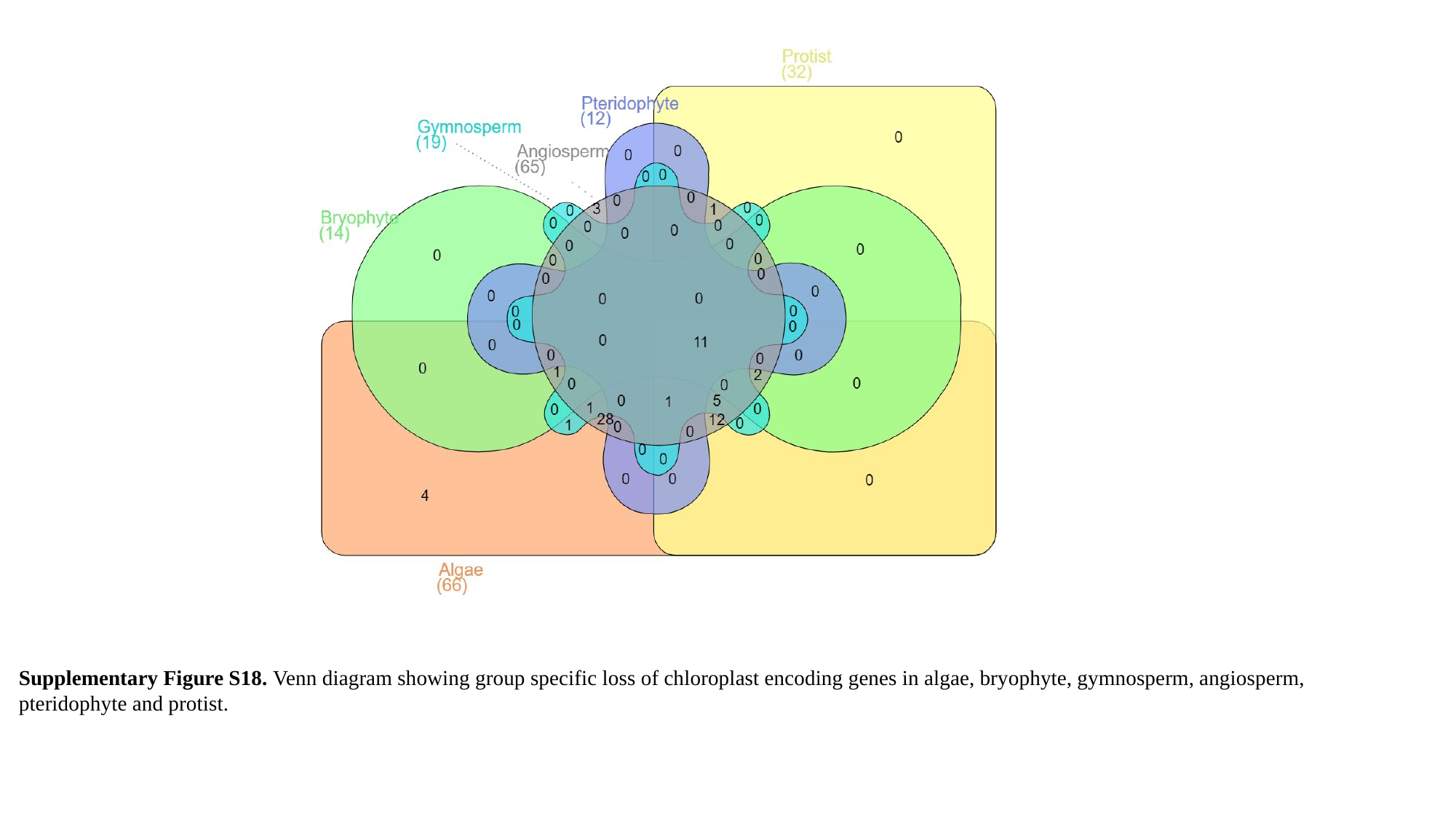

Supplementary Figure S18. Venn diagram showing group specific loss of chloroplast encoding genes in algae, bryophyte, gymnosperm, angiosperm, pteridophyte and protist.

## Slide 18
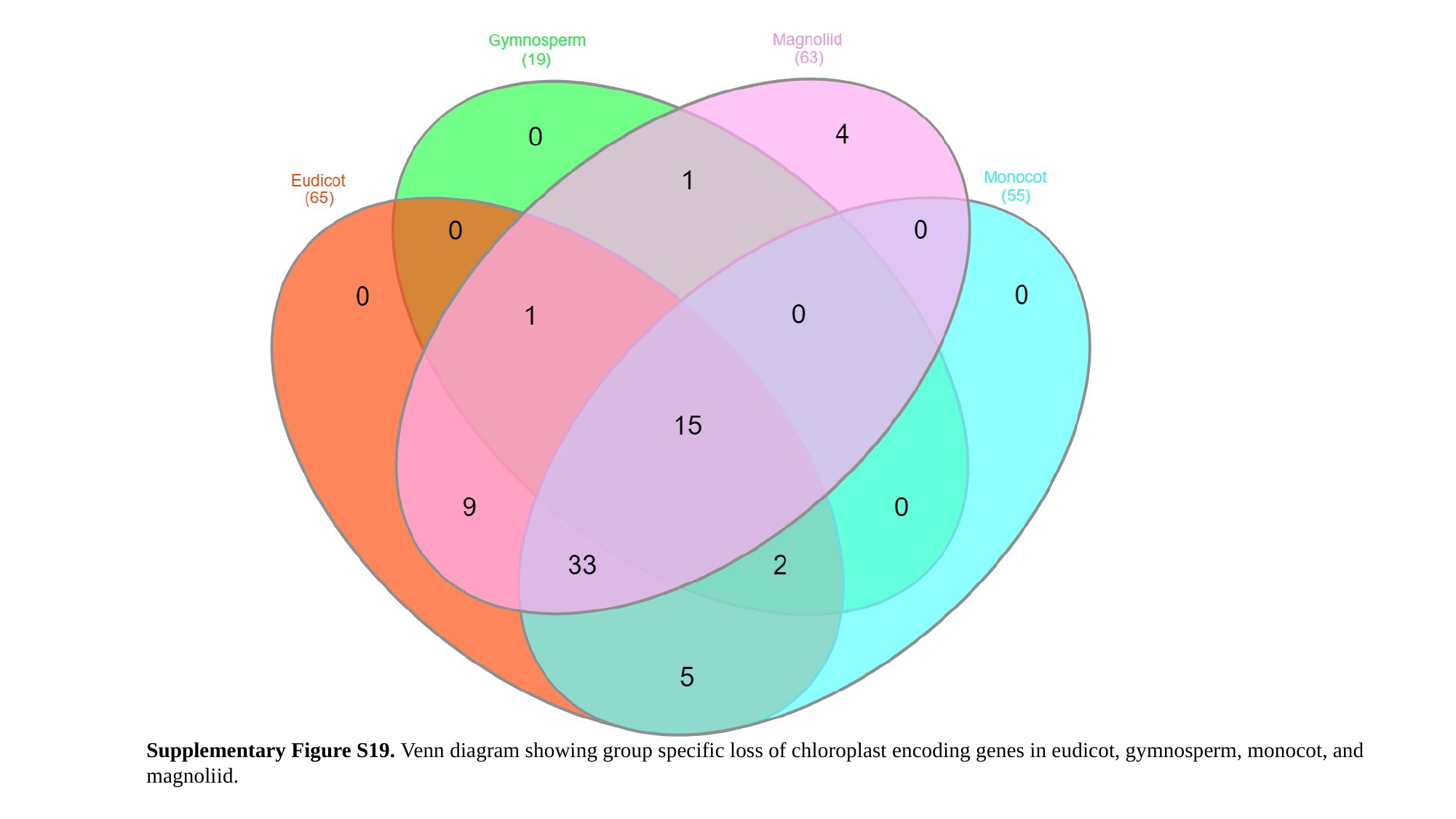

Supplementary Figure S19. Venn diagram showing group specific loss of chloroplast encoding genes in eudicot, gymnosperm, monocot, and magnoliid.
