# Supplementary material for: Gene Loss and Evolution of the Plastome
Source: Genes (Basel). 2020 Sep 25;11(10):1133. doi: 10.3390/genes11101133 (PMC7650654; doi:10.3390/genes11101133)
Supplement: Supplementary file 1 [file genes-11-01133-s001.zip › Supplementary Materials/Supplementary Table S1.docx]

**Supplementary Table 1**

Loss of chloroplast-encoding genes in different species of respective lineages.

| **Lineage** | **Lost genes** |
| --- | --- |
| Algae | accD, atpA, atpB, atpE, atpF, atpH, atpI, ccsA, cemA, clpP, ndhA, ndhB, ndhC, ndhD, ndhE, ndhF, ndhG, ndhH, ndhI, ndhJ, ndhK, petA, petB, petD, petG, petL, petN, psaA, psaB, psaC, psaI, psaJ, psbA, psbB, psbC, psbD, psbE, psbF, psbH, psbI, psbJ, psbK, psbL, psbT, psbZ, psbf1, rpl14, rpl16, rpl2, rpl22, rpl23, rpl32, rpl33, rpl36, rpoA, rpoB, rpoC2, rps12, rps14, rps15, rps16, rps18, rps2, rps3, rps4, rps7, Yc1, Ycf2, Ycf3, Ycf4 |
| Bryophytes | ccsA, ndhA, ndhC, ndhD, ndhE, ndhF, ndhG, ndhH, ndhI, ndhJ, ndhK, petN, rpoA, rps16 |
| Eudicots | accD, atpA, atpB, atpE, atpF, atpH, atpI, ccsA, cemA, clpP, ndhA, ndhB, ndhC, ndhD, ndhE, ndhF, ndhG, ndhH, ndhI, ndhJ, ndhK, petA, petB, petD, petG, petL, petN, psaA, psaB, psaC, psaI, psaJ, psbA, psbB, psbC, psbD, psbE, psbF, psbH, psbI, psbJ, psbK, psbL, psbT, psbZ, psbf1, rpl14, rpl2, rpl22, rpl23, rpl32, rpl33, rpl36, rpoA, rpoB, rpoC1, rpoC2, rps11, rps14, rps15, rps16, rps18, rps19, rps2, rps8, Ycf1, Ycf2, Ycf3, Ycf4 |
| Gymnosperms | accD, clpP, ndhA, ndhB, ndhC, ndhD, ndhE, ndhF, ndhG, ndhH, ndhI, ndhJ, ndhK, psaJ, rpl23, rpl32, rps15, rps16, rps7 |
| Magnoliids | accD, atpB, atpE, atpH, atpI, ccsA, cemA, clpP, ndhA, ndhB, ndhC, ndhD, ndhE, ndhF, ndhG, ndhH, ndhI, ndhJ, ndhK, petA, petB, petD, petG, petL, petN, psaA, psaB, psaC, psaI, psaJ, psbA, psbB, psbC, psbD, psbE, psbF, psbH, psbJ, psbL, psbZ, psbf1, rpl14, rpl16, rpl2, rpl22, rpl23, rpl32, rpl33, rpl36, rpoA, rpoB, rpoC1, rpoC2, rps11, rps12, rps14, rps18, rps19, rps2, rps3, rps4, rps7, rps8, Ycf1, Ycf2, Ycf3, Ycf4 |
| Monocots | accD, atpA, atpB, atpE, atpF, atpH, atpI, ccsA, cemA, ndhA, ndhB, ndhC, ndhD, ndhE, ndhF, ndhG, ndhH, ndhI, ndhJ, ndhK, petA, petB, petD, petG, petL, petN, psaA, psaB, psaC, psaI, psaJ, psbA, psbB, psbC, psbD, psbE, psbF, psbH, psbI, psbJ, psbK, psbL, psbT, psbZ, psbf1, rpl22, rpl23, rpl32, rpl33, rpoB, rpoC1, rpoC2, rps15, rps16, rps19, Ycf1, Ycf2, Ycf3, Ycf4 |
| Protists | accD, atpE, atpF, ccsA, cemA, clpP, ndhA, ndhB, ndhC, ndhD, ndhE, ndhF, ndhG, ndhH, ndhI, ndhJ, ndhK, petA, petD, petL, petN, psaI, psaJ, psbL, psbZ, rpl22, rpl32, rpl33, rps15, rps16, rps18, rps8, Ycf1, Ycf2, Ycf3, Ycf4 |
| Pteridophytes | ndhA, ndhB, ndhC, ndhD, ndhE, ndhF, ndhG, ndhH, ndhI, ndhJ, ndhK, rps16 |
