# Supplementary material for: Gene Loss and Evolution of the Plastome
Source: Genes (Basel). 2020 Sep 25;11(10):1133. doi: 10.3390/genes11101133 (PMC7650654; doi:10.3390/genes11101133)
Supplement: Supplementary file 1 [file genes-11-01133-s001.zip › Supplementary Materials/Supplementary Table S2.docx]

**Supplementary Table 2**

Maximum composite likelihood substitution of nucleotides. The entry reflects the probability of substitution (r) from one base (row) to another base (column). The rates of transitions are highlighted in bold and rates of transversion are highlighted in italics. The nucleotide frequencies (%) of A, T/U, G, and C for respective study are mentioned in the rows. The transition/transversion ratio are mentioned as K1 (purine) and K2 (pyrimidine). The transition/transversion bias R = [A*G*k1 + T*C*k2]/[(A+G) * (T+C)]. The codon position included were 1^st^ + 2^nd^ + 3^rd^ + non-coding. All the positions with less than 95% site coverage were eliminated. That is fewer than 5% alignment gaps. Missing data and ambiguous bases were allowed at any position. The C > T substitution is more frequent than T > C substitution and G > A substitution more frequently than A > G. The major mechanism mutation is deamination of 5’-methyl cytosine to uracil (thiamine) producing C > T or on the complementary stand G > A.

| **Lineages** |  | **A** | **T/U** | **C** | **G** | **K1** | **K2** |
| --- | --- | --- | --- | --- | --- | --- | --- |
| **Protist** | (A=36.07%, T/U=35.74%, C=14.32%, G=13.86%), Bias R=1.319 | | | | | 3.229 | 3.286 |
|  | **A** | - | 6.80 | 2.46 | **8.51** |  |  |
|  | **T/U** | 6.86 | - | **8.95** | 2.64 |  |  |
|  | **C** | 6.86 | **22.34** | - | 2.64 |  |  |
|  | **G** | **22.15** | 6.80 | 2.46 | - |  |  |
| **IR deletion** | (A=29.51%, T/U=31.37%, C=18.47%, G=20.65%) Bias R =1.512 | | | | | 2.951 | 3.42 |
|  | **A** | - | 6.05 | 3.56 | **11.75** |  |  |
|  | **T/U** | 5.69 | - | **12.18** | 3.98 |  |  |
|  | **C** | 5.69 | **20.69** | - | 3.98 |  |  |
|  | **G** | **16.8** | 6.05 | 3.56 | - |  |  |
| **Algae** | (A=35.89%, T/U=34.60%, C=14.45%, G=15.05%), Bias R = 1.307 | | | | | 3.009 | 3.279 |
|  | **A** | - | 6.73 | 2.81 | **8.81** |  |  |
|  | **T/U** | 6.98 | - | **9.22** | 2.93 |  |  |
|  | **C** | 6.98 | **22.07** | - | 2.93 |  |  |
|  | **G** | **21.01** | 6.73 | 2.81 | - |  |  |
| **Bryophytes** | (A=31.56%, T/U=32.69%, C=18.40%, G=17.34%), Bias R = 1.516 | | | | | 3.474 | 3.136 |
|  | **A** | - | 6.17 | 3.47 | **11.36** |  |  |
|  | **T/U** | 5.95 | - | **10.89** | 3.27 |  |  |
|  | **C** | 5.95 | **19.34** | - | 3.27 |  |  |
|  | **G** | **20.68** | 6.17 | 3.47 | - |  |  |
| (A=31.41%, T/U=31.54, C=18.38, G=18.66) Bias R=1.626 | | | | | | 3.618 | 3.352 |
| **Gymnosperms** | **A** | - | 5.75 | 3.35 | **12.31** |  |  |
|  | **T/U** | 5.73 | - | **11.24** | 3.4 |  |  |
|  | **C** | 5.73 | **19.28** | - | 3.4 |  |  |
|  | **G** | **20.72** | 5.75 | 3.35 | - |  |  |
| (A=30.90%, T/U=30.59, C=19.63, G=18.78), Bias R = 1.59 | | | | | | 3.343 | 3.379 |
| **Eudicot** | **A** | - | 5.71 | 3.66 | **11.71** |  |  |
|  | **T/U** | 5.78 | - | **12.37** | 3.5 |  |  |
|  | **C** | 5.78 | **19.28** | - | 3.5 |  |  |
|  | **G** | **19.33** | 5.71 | 3.66 | - |  |  |
| **Monocot** | (A=27.68%, T/U=33.16%, C=21.49%, G=17.66%) Bias R = 1.575 | | | | | 3.527 | 3.05 |
|  | A | - | 6.29 | 4.08 | **11.82** |  |  |
|  | T/U | 5.25 | - | **12.47** | 3.35 |  |  |
|  | C | 5.25 | **19.24** | - | 3.35 |  |  |
|  | G | **18.53** | 6.29 | 4.08 | - |  |  |
| (A=27.98%, T/U=29.69, C=22.19, G=2014) Bias R=2.15 | | | | | | 4.798 | 4.043 |
| **Pteridophytes** | A | - | 4.64 | 3.46 | **15.08** |  |  |
|  | T/U | 4.37 | - | **14.01** | 3.14 |  |  |
|  | C | 4.37 | **18.74** | - | 3.14 |  |  |
|  | G | **20.95** | 4.64 | 3.46 | - |  |  |
|  | (A=29.86%, T/U=31.39, C=19.97%, G=18.77%), Bias R = 1.548 | | | | | 3.357 | 3.165 |
| **Magnoliids** | A | - | 5.97 | 3.8 | **11.98** |  |  |
|  | T/U | 5.68 | - | **12.02** | 3.57 |  |  |
|  | C | 5.68 | **18.90** | - | 3.57 |  |  |
|  | G | **19.06** | 5.97 | 3.8 | - |  |  |
|  | A=29.46%, T/U=30.39%, C=20.48%, G=19.67%) Bias R=1.325 | | | | | 2.799 | 2.713 |
| **Nymphaeales** | A | - | 6.39 | 4.31 | **11.58** |  |  |
|  | T/U | 6.19 | - | **11.68** | 4.14 |  |  |
|  | C | 6.19 | **17.34** | - | 4.14 |  |  |
|  | G | **17.34** | 6.39 | 4.31 | - |  |  |
