# Supplementary figures and images for: Gene Loss and Evolution of the Plastome
Source: Genes (Basel). 2020 Sep 25;11(10):1133. doi: 10.3390/genes11101133 (PMC7650654; doi:10.3390/genes11101133)

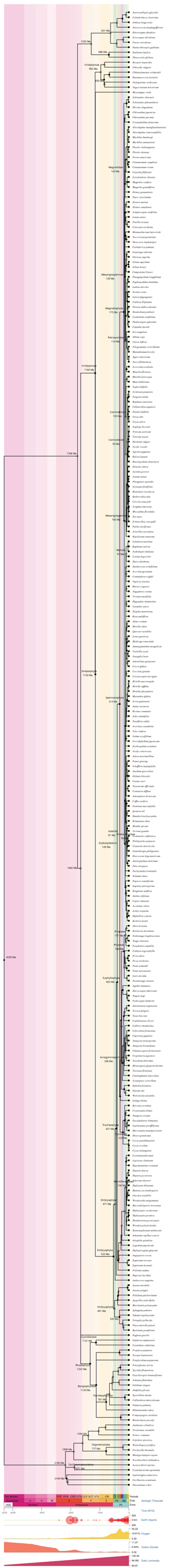

Supplement: Supplementary file 1 [file genes-11-01133-s001.zip › Supplementary Materials/Supplementary Figure S20.tif]
